# Amination of Heteroaryl Chlorides: Palladium Catalysis or S_N_Ar in Green Solvents?

**DOI:** 10.1002/cssc.201300239

**Published:** 2013-06-21

**Authors:** Katie Walsh, Helen F Sneddon, Christopher J Moody

**Affiliations:** [a] School of Chemistry, University of NottinghamUniversity Park, Nottingham NG7 2RD (UK), Fax: (+44) 115 951 3564; [b] Green Chemistry Performance Unit, GlaxoSmithKline R…D Ltd, Medicines Research CentreStevenage, Hertfordshire SG1 2NY (UK)

**Keywords:** amination, aromatic substitution, green chemistry, nucleophilic substitution

## Abstract

The reaction of heteroaryl chlorides in the pyrimidine, pyrazine and quinazoline series with amines in water in the presence of KF results in a facile S_N_Ar reaction and *N*-arylation. The reaction is less satisfactory with pyridines unless an additional electron-withdrawing group is present. The results showed that the transition-metal-free S_N_Ar reaction not only compares favourably to palladium-catalysed coupling reactions but also operates under environmentally acceptable (“green”) conditions in terms of the base and solvent.

## Introduction

**Figure 1:**
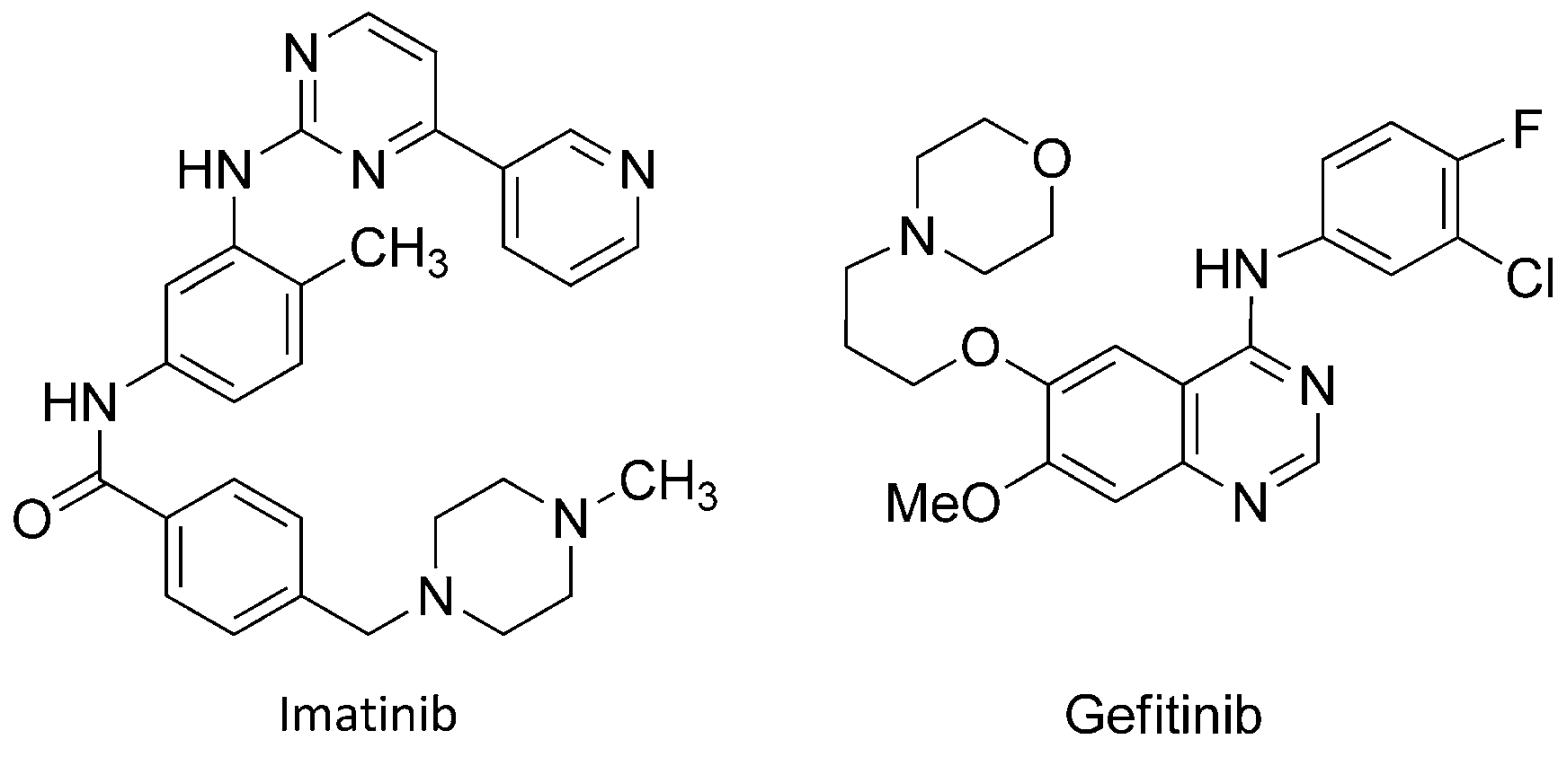
Structures of Imatinib and Gefitinib.

The formation of aryl C–N bonds is a fundamental process within organic chemistry, and the product *N*-arylamines are present in various natural products and pharmaceutical molecules.[1] Examples include the well-known clinically used kinase inhibitors Imatinib and Gefitinib (Figure 1). Recent analyses showed heteroatom alkylations and arylations to be the largest single class of transformations used in medicinal chemistry, and heteroaryl *N*-arylations make up a significant subclass of these transformations.^[^2^,^^3^^]^ Classically, these structures have been accessed through nucleophilic aromatic substitution (S_N_Ar) reactions on appropriately activated substrates,[4] although poor substrate scope and reactivity are major limitations to this method. Alternatively, the copper-mediated amination of halobenzenes, discovered by Ullmann in 1903 and shown to be catalytic by Goldberg three years later, can also be used for the synthesis of a range of *N*-aryl compounds, and although the original methodology had limitations, recent developments have resulted in substantial improvements.[5] However, the most significant breakthrough in this area came in 1994 when Buchwald et al. and Hartwig et al. independently developed a palladium-catalysed *N*-arylation reaction.^[^6^,^^7^^]^ With its improved substrate scope and functional group tolerance, the Buchwald–Hartwig amination has become a fundamental part of modern organic chemistry.[8]

**Scheme 1:**
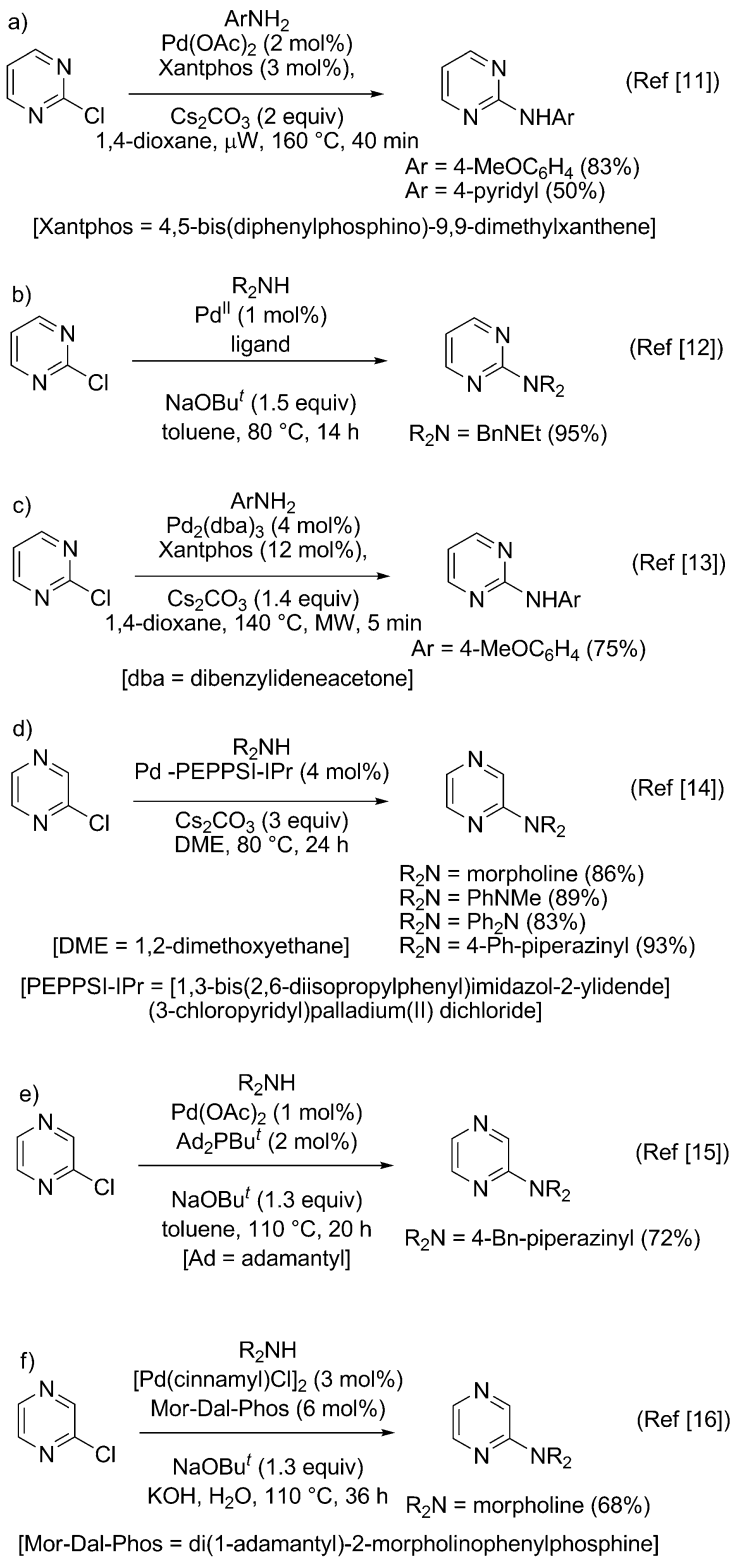
Examples of palladium-catalysed amination of reactive heteroaryl chlorides.

However, precious metals are expensive and dwindling resources, and although the success of palladium-catalysed reactions has revolutionised *N*-arylation chemistry, there is a risk that they are used without due consideration of alternatives. For instance, 2-chloropyrimidine is 10^14^–10^16^ times more reactive than chlorobenzene in terms of its ability to undergo S_N_Ar reactions;^[^9,10^]^ however, an examination of the recent literature reveals that even such reactive substrates are subjected to palladium-catalysed amination reactions. Some recent examples of the palladium-catalysed amination of 2-chloropyrimidine and chloropyrazine are shown in Scheme 1,^[^11^–^16^]^ and although these reactions, some of which are performed under fairly forcing conditions with non-trivial ligands, proceed in good yield, it does beg the question as to whether palladium is really needed for such highly activated substrates. The literature contains many examples of activated heteroaromatic chlorides reacting readily under S_N_Ar conditions, so the fact that such processes appear to have been side-lined in favour of their palladium-mediated counterparts puzzled us. Thus, we sought to optimise the coupling of heteroaromatic chlorides with amine nucleophiles under S_N_Ar conditions with a view to defining parameters that not only resulted in comparable yields to those given by the published palladium-catalysed methods but also operated under environmentally acceptable conditions in terms of the base and solvent. We now report the results of this detailed study.

## Results and Discussion

The reaction of chloropyrazine with a secondary amine morpholine to yield morpholinopyrazine (**1**) was taken as a simple test reaction to screen a range of solvents and bases, although from the outset we limited ourselves to solvents that are generally accepted as “green”.[17] Solvents with environmental or toxicity alerts were disregarded, and as a result of previous literature,^[^11^,^^14^^]^ caesium carbonate was chosen initially as the base for these reactions. Although caesium carbonate sometimes creates problematic waste streams on large scale, its solubility made it a good starting point for these studies. Organic solvents were found to be generally ineffective for chloropyrazine reactions (Table 1, entries 1–6), although they are better in the reaction of 2-chloropyrimidine and yield 2-morpholinopyrimidine (**2**) (data shown in the Supporting Information). However, for both the chloroheterocycles, the best result was obtained by using water as a solvent, which gave both the highest yield and the cleanest reaction mixtures; in most cases, the product required only a simple extraction with isopropyl acetate. Because the reaction mixture is not homogeneous, this unexpected result could be attributed to an “on-water” effect.[18] Yields were improved from 33% to 58 % (Table 1, entry 6) by increasing the temperature to 100 °C. Water is not automatically a green solvent. For any given reaction, consideration needs to be given to how wastewater is to be handled—whether it is energy intensive to clean or whether contaminated aqueous streams have to be incinerated in which other waste solvent streams could be recycled or productively burnt to generate heat and power. However, if multiple factors are weighed,[17] water is often one of the more benign choices. Isopropyl acetate was chosen for extractions on the basis that it can be easier to recover and recycle on a large scale than ethyl acetate.

**Table 1:** Reaction of 2-chloropyrazine with morpholine with various solvents and bases.[a]
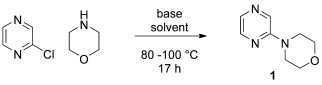

Entry	Base	Solvent	Yield [%]
1	Cs_2_CO_3_	2-Me-THF	3
2	Cs_2_CO_3_	2-Me-THF/H_2_O (1:1)	18
3	Cs_2_CO_3_	1-butanol	<5
4	Cs_2_CO_3_	EtOAc	4
5	Cs_2_CO_3_	H_2_O	33[b]
6	Cs_2_CO_3_	H_2_O	58
7	DBU[c]	H_2_O	40[b]
8	Et_3_N	H_2_O	38
9	K_2_CO_3_	H_2_O	63
10	CaCO_3_	H_2_O	38
11	K_3_PO_4_	H_2_O	60
12	KF	H_2_O	70
13	KF[d]	H_2_O	30
14	KF[e]	H_2_O	64[b]
15	KF	H_2_O	47[b]
16	KF	H_2_O	63^f^
17	KF	H_2_O	81^[^d^,^^g^^]^

[a] All reactions were performed with chloropyrazine (1 equiv.), morpholine (1 equiv.) and base (2 equiv.) in the specified solvent at 80 °C for 17 h. Reactions in water were performed at 100 °C unless otherwise specified.

[b] The reaction was performed at 80 °C.

[c] DBU=1,8-diazabicycloundec-7-ene.

[d] The reaction used 1 equiv. of base.

[e] The reaction used 3 equiv. of base.

[f] The reaction was performed in a microwave reactor (300 W) at 150 °C for 30 min.

[g] The reaction was performed in a microwave reactor (300 W) at 175 °C for 30 min.

We then investigated the reactions of chloropyrazine with morpholine in water with respect to the base (Table 1, entries 7–15). Organic bases proved inefficient in water (entries 7 and 8), and moderate yields were obtained with other carbonates or potassium phosphate bases (entries 9–11). Potassium fluoride was found to be the most effective base and was thus investigated further. Reducing the amount of base to 1 equiv. dramatically lowered the yield (entry 13); however, only a small increase in yield was observed by increasing the amount to 3 equiv. (entry 14). However, with 2 equiv. of potassium fluoride in water at reflux (entry 12), the highest yield of 70 % of **1** was achieved. Reaction times were reduced dramatically by performing the reaction in a microwave reactor for 30 min at 150 or 175 °C (63 % and 81 % yield, respectively). Inductively coupled plasma mass spectrometry (ICP-MS) analysis was performed to determine what levels of other metals were present in the sample of potassium fluoride. Copper could not be detected at a limit of 24 ppb, and palladium was not detected at a limit of 10 ppb.

These results are favourably comparable to those obtained with the palladium-catalysed variants that produced **1** in 86 % yield with Pd-PEPPSI, Cs_2_CO_3_ in 1,2-dimethoxyethane (DME) at 80 °C (Scheme 1d)[14] or in 68 % yield with [Pd(cinnamyl)Cl]_2_, Mor-Dal-Phos, NaOBu^*t*^, KOH, and H_2_O at 110 °C (Scheme 1e).[16] However, other researchers have also found that the palladium catalyst is unnecessary and the reaction of chloropyrazine with morpholine proceeds at 130 °C in DMSO.[19]

**Figure 2:**
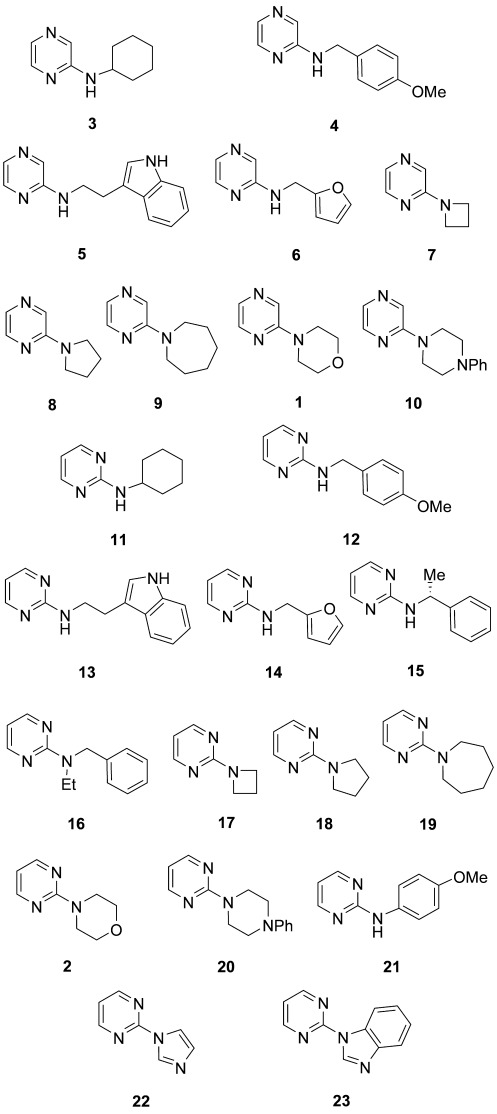
Amination products of chloropyrazine and 2-chloropyrimidine.

With the useful KF-in-water conditions now established, chloropyrazine and 2-chloropyrimidine were screened against a wide range of primary and secondary amines as well as anilines and NH heteroaromatic compounds; they were selected because they contain a range of functional groups that are relevant to contemporary medicinal chemistry and yielded the corresponding *N*-arylamines **1**–**23** (Table 2 and Figure 2). As expected, reactions with the more reactive 2-chloropyrimidine generally produced higher yields (2-chloropyrimidine is ≈100 times more reactive than chloropyrazine)[9] and reacted in moderate to excellent yield with primary and secondary amines (Table 2, entries 1–11). In the case of α-methylbenzylamine (entry 5), HPLC demonstrated that there was no loss of enantiomeric excess in the final product (*ee*>98). With *p*-anisidine (entry 12), the yield of 86 % was comparable with that obtained through the corresponding palladium-catalysed amination [Pd(OAc)_2_, Xantphos, dioxane, microwave, 160 °C, 83 %; Scheme 1a].[11] However, the reaction was poor with *ortho*-substituted anilines and 2-aminothiazole not reacting. Chloropyrazine gave moderate to excellent yields with electron-rich primary and secondary amines, but was unreactive with all anilines and NH heterocycles examined. Reaction times can be reduced again by conducting the reaction in a microwave reactor for 60 min at 175 °C (entries 1 and 4). The structures of the products **1**–**23** of amination of chloropyrazine and 2-chloropyrimidine with the range of amines are shown in Figure 2.

**Table 2:** Amination of chloropyrazine and 2-chloropyrimidine.[a]
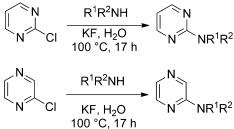

Entry	Amine	Product (yield [%]) for the reaction with	
		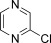	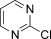
1	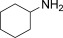	**3** (28; MW 60[c])	**11** (77)
2	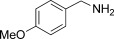	**4** (47)	**12** (85)
3	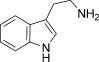	**5** (58)	**13** (85)
4	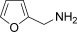	**6** (33; MW 52[c])	**14** (81)
5	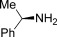	–	**15** (78)
6	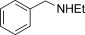	**–**	**16** (80) [95]^[b]^
7	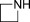	**7** (48)	**17** (52)
8	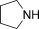	**8** (76)	**18** (76)
9	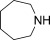	**9** (52)	**19** (69)
10	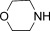	**1** (70) [68, 86][b]	**2** (84)
11	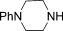	**10** (81[d]) [93][b]	**20** (93)
12	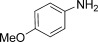	no reaction	**21** (86) [75, 83][b]
14	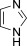	no reaction	**22** (62)
15	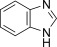	no reaction	**23** (83)

[a] All reactions were performed with heteroaryl halide (1 equiv.), amine (1 equiv.) and KF (2 equiv.) in water at 100 °C for 17 h.

[b] Yields in square brackets refer to the palladium-catalysed variants shown in Scheme 1.

[c] MW refers to the yield obtained if the reaction was performed in a microwave reactor (300 W) at 175 °C for 60 min with KF (1 equiv.).

[d] Yield reported as an NMR yield calculated from the internal standard using 1,4-dioxane.

In the case in which direct comparison is possible, our amination method involving KF in water can be compared with the palladium-catalysed protocols outlined in Scheme 1 (Table 2, entries 6 and 10–12): 80 % vs. 95 %, 70 % vs. 68 or 86 %, 81 % vs. 93 % and 86 % vs. 75 or 83 %. In addition, the coupling of 2-chloropyrimidine with imidazole and benzimidazole performed under copper catalysis resulted in 90 % and 100 % yield, respectively,[20] which are comparable to the slightly poorer yields of 62 % and 83 % obtained under our conditions (Table 2, entries 14 and 15). In other cases, the transition-metal catalyst is clearly beneficial. Although chloropyrazine does not react readily with 4-methoxyaniline under our S_N_Ar conditions (Table 2, entry 12), palladium catalysis with the BrettPhos [2-(dicyclohexylphosphino)3,6-dimethoxy-2′,4′,6′-triisopropyl-1,1′-biphenyl] ligand results in a high yield of the coupled product.[21] Given the high reactivity of 2-chloropyrimidine towards nucleophilic attack, it is not surprising that there are other examples of the S_N_Ar process involving amine nucleophiles (pyrrolidine,[22] cyclohexylamine and 4-methoxybenzylamine),[13] which in combination with our own results reinforce the idea that precious metal catalysis is not needed for amination reactions with such highly activated heteroaryl halides.

**Figure 3:**
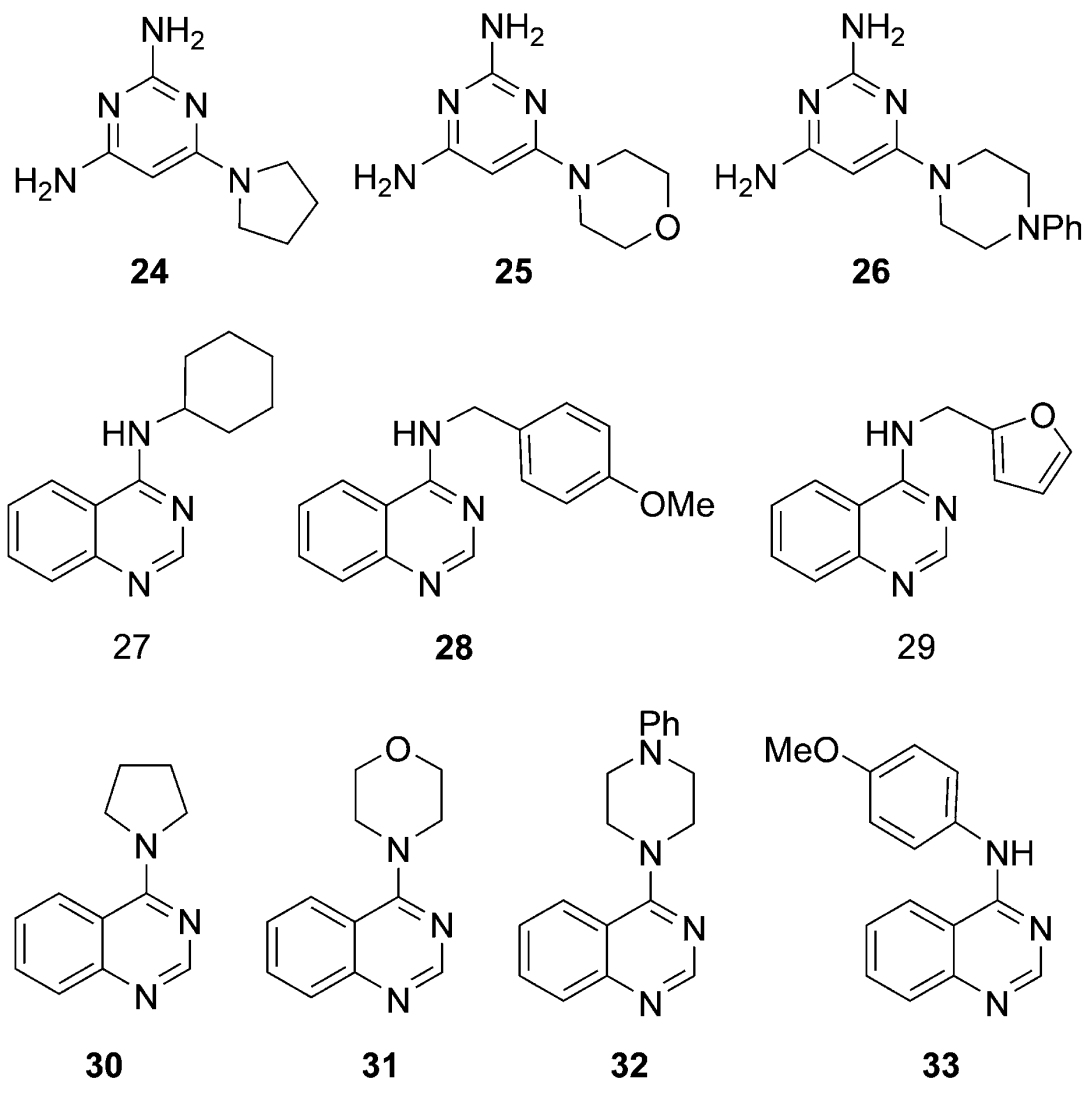
Amination products of halopyrimidines and 4-chloroquinazoline.

From this list of amines, seven examples were chosen to be tested against other pyrimidines in comparison to 2-chloropyrimidine (Table 3). Unsurprisingly, 2-bromopyrimidine showed similar reactivity; however, the reactions with 4-chloro-2,6-diaminopyrimidine resulted in unpredictable yields, possibly as a result of solubility issues. 4-Chloroquinazoline gave good to excellent yields in all cases, which is in line with its well-known reactivity in S_N_Ar reactions, for example in the synthesis of 4-anilinoquinazolines that are used as kinase inhibitors.[23] The structures of the new products **24**–**33** obtained are shown in Figure 3.

**Table 3:** Amination of halopyrimidines and 4-chloroquinazoline.[a]
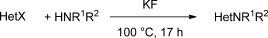

Entry	Amine		Product (yield [%]) for the reaction with		
		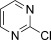	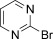	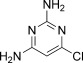	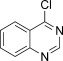
1	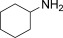	**11** (77)	**11** (72)	no reaction	**27** (78)
2	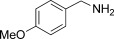	**12** (85)	**12** (89)	no reaction	**28** (71)
3	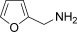	**14** (81)	**14** (68)	no reaction	**29** (71)
4	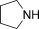	**18** (76)	**18** (59)	**24** (45)	**30** (97)
5	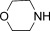	**2** (84)	**2** (85)	**25** (49)	**31** (80)
6	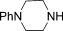	**20** (93)	**20** (91)	**26** (80)	**32** (82)
7	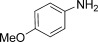	**21** (86)	**21** (65)	no reaction	**33** (86)

[a] All reactions were performed with aryl halide (1 equiv.), amine (1 equiv.) and KF (2 equiv.) in water at 100 °C for 17 h.

**Figure 4:**
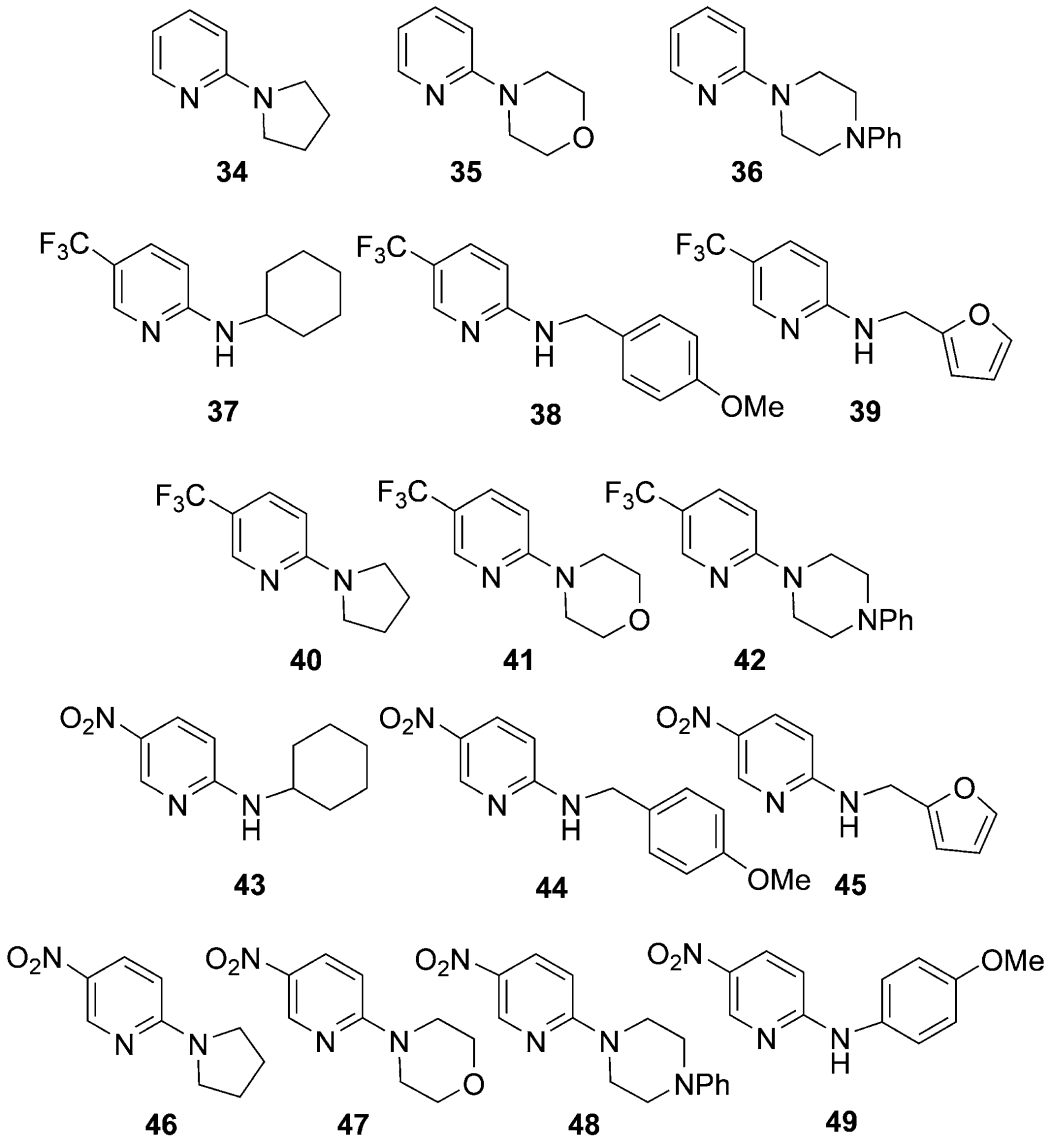
Amination products of 2-halopyridines.

The same seven representative amines were also reacted with halopyridines under the KF/water conditions (Table 4), even though 2-chloropyridine is approximately 10^8^ times less reactive towards nucleophiles than 2-chloropyrimidine under S_N_Ar conditions. Hence, the reactions of the amines with 2-chloropyridine were generally unsatisfactory, although, as expected for an S_N_Ar reaction, 2-fluoropyridine resulted in better yields. If the pyridine substrate contained an additional electron-withdrawing group such as trifluoromethyl or nitro, these substituted pyridines demonstrated reactivities similar to pyrimidines. 2-Chloro-5-nitropyridine gave good results with all the amines examined. The structures of the wide range of *N*-pyridylamines **34**–**49** obtained are shown in Figure 4.

**Table 4:** Amination of 2-halopyridines.[a]
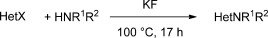

Entry	Amine		Product (yield [%]) for the reaction with		
		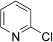	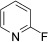	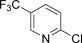	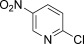
1	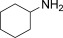	(<5)	(6)	**37** (60)	**43** (96)
2	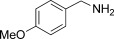	(<5)	no reaction	**38** (65)	**44** (70)
3	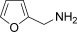	(<5)	no reaction	**39** (48; MW 59)[b]	**45** (73)
4	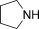	**34** (<5)	**34** (54)	**40** (53)	**46** (74)
5	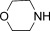	**35** (9; MW 25)[b]	**35** (16)	**41** (36)	**47** (87)
6	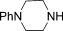	**36** (21)[c]	**36** (46)[c]	**42** (85)	**48** (76)
7	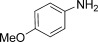	no reaction	no reaction	no reaction	**49** (73)

[a] All reactions were performed with aryl halide (1 equiv.), amine (1 equiv.) and KF (2 equiv.) in water at 100 °C for 17 h.

[b] MW refers to the yield if the reaction was performed in a microwave reactor (300 W) at 175 °C for 1 h (2 h in the case of furfurylamine) with KF (1 equiv.).

[c] Yield reported as an NMR yield calculated from the internal standard using 1,4-dioxane.

Although 2-bromopyridine is reported to react with pyrrolidine under microwave irradiation,[22] it is clear that in the absence of additional activation, 2-halopyridines represent the limit of what will undergo facile amination reactions under our simple S_N_Ar conditions. In contrast, both 2-bromo- and 2-chloropyridine undergo coupling under a range of palladium-catalysed conditions with, for example, cyclohexylamine and pyrrolidine,[21] with morpholine[12], [16] and with 4-methoxyaniline (63 %).[24]

## Conclusions

Although palladium-catalysed *N*-arylation amination reactions have undoubtedly made a major impact on synthetic organic chemistry, there are examples of reactions involving activated halides, in which it could appear that the use of transition metals was unnecessary. We have addressed this issue of over-reliance on palladium catalysis in organic chemistry in a range of nucleophilic aromatic substitution reactions with activated heteroaryl halides to synthesise various heteroaryl amine substrates. A set of conditions has been developed with potassium fluoride and water at reflux for 17 h that allow for S_N_Ar chemistry to be performed, without the use of palladium catalysis and with various heteroaryl halides and amines. The results showed that the transition-metal-free S_N_Ar reaction not only compares favourably to palladium-catalysed coupling reactions but also operates under environmentally acceptable conditions in terms of the base and solvent.

Whilst we believe that this methodology offers an improvement over many Pd-catalysed reactions, the compatibility of fluoride ions with other reagents and waste streams (on a large scale) should be considered.

## Experimental Section

General Procedure: To a Reacti-Vial (Thermo Scientific, 5 mL) was added aryl halide (1.75 mmol), amine (1.75 mmol), potassium fluoride (3.50 mmol) and the solvent (1 mL); the resulting mixture was heated to 100 °C for 17 h on a heating block. Upon cooling, the mixture was quenched with an aqueous potassium carbonate solution (40 mL) and extracted into isopropyl acetate (2×30 mL). The organic extracts were then combined and washed with brine before they were dried over sodium sulfate and the solvent evaporated under reduced pressure. If necessary, purification was performed by using column chromatography over silica gel (light petroleum/ethyl acetate 4:1).

## Notes

As a service to our authors and readers, this journal provides supporting information supplied by the authors. Such materials are peer reviewed and may be re-organized for online delivery, but are not copy-edited or typeset. Technical support issues arising from supporting information (other than missing files) should be addressed to the authors.
