# Supplementary material for: Amination of Heteroaryl Chlorides: Palladium Catalysis or SNAr in Green Solvents?
Source: ChemSusChem. 2013 Jun 21;6(8):1455–60. doi: 10.1002/cssc.201300239 (PMC3792620; doi:10.1002/cssc.201300239)
Supplement: Supplementary file 1 [file CSSC0006-1455-SD1.PDF]

CHEMISTRY & SUSTAINABILITY
CHEMSUSCHEM
ENERGY & MATERIALS

Supporting Information

© Copyright Wiley-VCH Verlag GmbH & Co. KGaA, 69451 Weinheim, 2013

Amination of Heteroaryl Chlorides: Palladium Catalysis or S_NAr in Green Solvents?

Katie Walsh,[a] Helen F. Sneddon,[b] and Christopher J. Moody*[a]

cssc_201300239_sm_miscellaneous_information.pdf

Experimental Section

General information

Commercially available reagents were used throughout without purification unless otherwise stated. All anhydrous solvents were used as supplied, except tetrahydrofuran and dichloromethane that were freshly distilled according to standard procedures. Reactions were routinely carried out under an argon atmosphere unless otherwise stated, and all glassware was flame-dried before use. Light petroleum refers to the fraction with bp 40-60 ºC. Ether refers to diethyl ether.

Analytical thin layer chromatography was carried out on aluminium backed plates coated with silica gel, and visualised under UV light at 254 and/or 360 nm and/or by chemical staining. Flash chromatography was carried out using silica gel, with the eluent specified. Infrared spectra were recorded using an FT-IR spectrometer over the range 4000-600 cm^{-1}. NMR spectra were recorded at 400 MHz (^{1}H frequency, 100 MHz ^{13}C frequency). Chemical shifts are quoted in parts per million (ppm), and are referenced to residual H in the deuterated solvent as the internal standard. Coupling constants, *J*, are quoted in Hz. In the ^{13}C NMR spectra, signals corresponding to CH, CH_2, or CH_3 groups are assigned from DEPT. Mass spectra were recorded on a time-of-flight mass spectrometer using electrospray ionisation (ESI), or an EI magnetic sector instrument.

General Procedure Method A

To a 5 mL Reacti-vial (Thermo Scientific) was added aryl halide (1.75 mmol), amine (1.75 mmol), potassium fluoride (3.50 mmol) in solvent (1 mL) and the resulting mixture heated to 100 °C for 17 h on a heating block. Once cooled, the mixture was quenched with aqueous potassium carbonate solution (40 mL) and extracted into isopropyl acetate (2 × 30 mL). The organic extracts were then combined and washed with brine before being dried over sodium

sulfate and the solvent evaporated under reduced pressure. When necessary, purification was carried out by column chromatography over silica gel (light petroleum/ ethyl acetate, 4:1).

General Procedure Method B

To a microwave vial was added aryl halide (1.75), amine (1.75 mmol), base (3.50 mmol) in water (1 mL) and the mixture heated to 175 °C for 30-120 min in a CEM Discover^{TM} S-class (300W) microwave. Once cooled, the mixture was quenched with aqueous potassium carbonate solution (40 mL) and extracted into isopropyl acetate (2 × 30 mL). The organic extracts were then combined and washed with brine before being dried over sodium sulfate and the solvent evaporated under reduced pressure. When necessary, purification was carried out by column chromatography over silica gel (light petroleum/ ethyl acetate, 4:1).

4-(Pyrazin-2-yl)morpholine 1

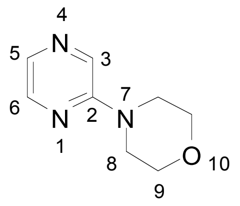

The title compound was synthesised following general procedure method A from 2-chloropyrazine (156 μL, 1.75 mmol), morpholine (153 μL, 1.75 mmol) and potassium fluoride (203 mg, 3.50 mmol) in water (3 mL) at 100 °C for 17 h to give the *title compound* a pale yellow crystalline solid (199 mg, 70%); mp 48-50 °C (lit.,[1] mp 46-48 °C); (Found; MH^+, 166.0979. $C_8H_{12}N_3O^+$ requires 166.0975); ν_{max}/cm^{-1} (ATR) 2975, 2868, 1581, 1519, 1483, 1427; δ_H (400 MHz; $CDCl_3$) 8.13 (1 H, d, *J* 1.5, 3-H), 8.08 (1 H, dd, *J* 2.6, 1.5, 5-H), 7.90 (1 H, d, *J* 2.6, 6-H) 3.86 (4 H, t, *J* 4.8, 9-H) 3.58 (4 H, t, *J* 4.8, 8-H); δ_C (75 MHz; $CDCl_3$) 155.1 (C), 141.8 (CH), 133.6 (CH), 130.9 (CH), 66.5 (CH_2), 44.8 (CH_2).

4-(Pyrimidin-2-yl)morpholine 2

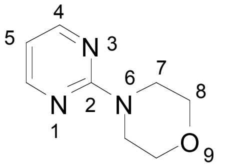

The title compound was synthesised following general procedure method A from 2-chloropyrimidine (200 mg, 1.75 mmol), morpholine (153 μL, 1.75 mmol) and potassium fluoride (203 mg, 3.50 mmol) in water (1 mL) at 100 °C for 17 h to give the title compound as a yellow oil (243 mg, 84%); (Found; MH^+, 166.0975. $C_8H_{12}N_3O^+$ requires 166.0975); ν_{max}/cm^{-1} ($CDCl_3$) 3032, 2861, 1586, 1551, 1446, 1357; δ_H (400 MHz; $CDCl_3$) 8.34 (2 H, d, *J* 4.8, 4-H), 6.55 (1 H, t, *J* 4.8, 5-H), 3.84-3.77 (8 H, m, 7,8-C); δ_C (75 MHz; $CDCl_3$) 161.8 (C), 157.7 (CH), 110.3 (CH), 66.8 (CH_2), 44.2 (CH_2). Data recorded matches literature.[2]

***N*-Cyclohexylpyrazin-2-amine 3**

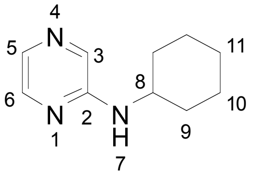

The title compound was synthesised following general procedure method A from 2-chloropyrazine (156 μL, 1.75 mmol), cyclohexylamine (201 μL, 1.75 mmol), potassium fluoride (203 mg, 3.50 mmol) in water (1 mL) at 100 °C for 17 h the give the *title compound* as a cream solid (87 mg, 28%); mp 106-108 °C; (Found; MH^+, 178.1348. $C_{10}H_{15}N_3^+$ requires 178.1339); ν_{max}/cm^{-1} (ATR) 3258, 2925, 2848, 1593, 1510, 1443; δ_H (400 MHz; $CDCl_3$) 7.97 (1 H, dd, *J* 2.8, 1.5, 5-H), 7.86 (1 H, d, *J* 1.5, 3-H), 7.77 (1 H, d, *J* 2.8, 6-H), 4.49 (1 H, bs, NH), 3.69 (1 H, tdt, *J* 10.4, 7.9, 3.9, 8-H), 2.11-2.03 (2 H, m, CH_2), 1.83-1.75 (2H, m, CH_2), 1.71-1.64 (1H, m, CH_2), 1.49-1.39 (2H, m, CH_2), 1.31-1.19 (3H, m, CH_2); δ_C (75 MHz;

$CDCl_3$) 154.0 (C), 142.0 (CH), 132.3 (CH), 132.1 (CH), 49.7 (CH), 33.2 (CH_2), 25.7 (CH_2), 24.8 (CH_2).

N-(4-Methoxybenzyl)pyrazin-2-amine 4

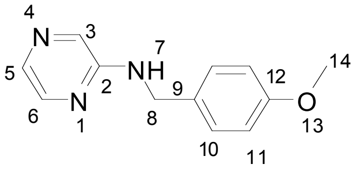

The title compound was synthesised following general procedure method A from 2-chloropyrazine (156 μL, 1.75 mmol), 4-methoxybenzylamine (229 μL, 1.75 mmol) and potassium fluoride (203 mg, 3.50 mmol) in water (1 mL) at 100 °C for 17 h to give the *title compound* as a colourless solid (268 mg, 47%); mp 97-99 °C; (Found; MH^+, 216.1132. $C_{12}H_{14}N_3O^+$ requires 216.1131); ν_{max}/cm^{-1} (ATR) 3229, 2963, 1584, 1526, 1509, 1468; δ_H (400 MHz; $CDCl_3$) 8.03 (1H, dd, *J* 2.8, 1.5, 5-H), 7.91 (1H, d, *J* 1.5, 3-H), 7.85 (1H, d, *J* 2.8, 6-H), 7.31 (2H, d, *J* 8.7, ArH), 6.91 (2H, d, *J* 8.7, ArH), 4.87 (1H, bs, NH), 4.51 (2H, d, *J* 5.6, 8-H), 3.83 (3H, s, CH_3); δ_C (75 MHz; $CDCl_3$) 159.1 (C), 154.5 (C), 142.0 (CH), 133.0 (CH), 132.1 (CH), 130.5 (C), 129.0 (CH), 114.2 (CH), 55.3 (CH_3), 45.1 (CH_2).

N-(2-(1H-indol-2-yl)ethyl)pyrazin-2-amine 5

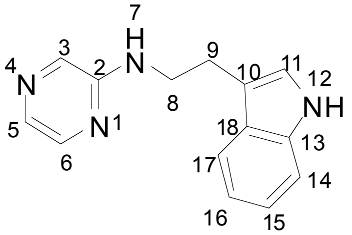

The title compound was synthesised following general procedure method A from 2-chloropyrazine (156 μL, 1.75 mmol), tryptamine (280 mg, 1.75 mmol) and potassium fluoride (203 mg, 3.50 mmol) in water (1 mL) at 100 °C for 17 h to give the *title compound* as a

colourless oil (242 mg, 58%); mp 135-137 °C (Found; MH^+, 239.1294. $C_{14}H_{15}N_4^+$ requires 239.1291); ν_{max}/cm^{-1} ($CHCl_3$) 3691, 3606, 3479, 3062, 3005, 1588, 1537, 1505; δ_H (400 MHz; $CDCl_3$) 8.27 (1H, bs, NH), 8.01 (1H, dd, *J* 2.7, 1.4, 5-H), 7.83 (1H, d, *J* 1.4, 3-H), 7.81 (1H, d, *J* 2.7, 6-H), 7.64 (1H, d, *J* 7.9, ArH), 7.39 (1H, d, *J* 8.2, ArH), 7.23 (1H, t, *J* 8.2, ArH), 7.15 (1H, t, *J* 7.9, ArH), 7.06 (1H, d, *J* 2.4, 11-H), 4.73 (1H, bs, NH), 3.73 (2H, q, *J* 6.6, 8-H), 3.12 (2H, t, *J* 6.6, 9-H); δ_C (75 MHz; $CDCl_3$) 154.6 (C), 142.0 (CH), 136.5 (C), 132.6 (CH), 132.3 (CH), 127.3 (C), 122.3 (CH), 122.2 (CH), 119.5 (CH), 118.7 (Ar-C), 112.9 (C), 111.3 (CH), 41.5 (CH_2), 25.1 (CH_2).

_N_-(Fur-2-ylmethyl)pyrazin-2-amine 6

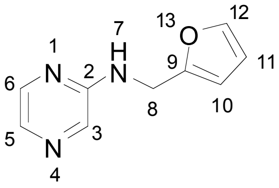

The title compound was synthesised following general procedure method A from 2-chloropyrazine (156 μL, 1.75 mmol), furfurylamine (155 μL, 1.75 mmol) and potassium fluoride (203 mg, 1.75 mmol) in water (1 mL) at 100 °C for 17 h to give the *title compound* as a brown oil (102 mg, 33%); (Found; MH^+, 176.0817. $C_9H_{10}N_3O^+$ requires 176.0818); ν_{max} (ATR)/cm^{-1} 3155, 2092, 1466, 1382, 1096, 928; δ_H (400 MHz; $CDCl_3$) 8.04 (1H, dd, *J* 2.8, 1.5, 5-H), 7.96 (1H, d, *J* 1.5, 3-H), 7.87 (1H, d, *J* 2.8, 6-H), 7.39 (2H, dd, *J* 1.9, 1.0, ArH), 6.36 (1H, dd, *J* 3.2, 1.9, ArH), 6.29 (1H, dd, *J* 3.2, 1.0, ArH), 4.91 (1H, bs, NH), 4.59 (2H, d, *J* 5.5, 8-H); δ_C (75 MHz; $CDCl_3$) 154.1 (C), 151.8 (C), 142.2 (CH), 141.9 (CH), 133.4 (CH), 132.5 (CH), 110.4 (CH), 107.4 (CH), 38.5 (CH_2).

2-(Azetidin-1-yl)pyrazine 7

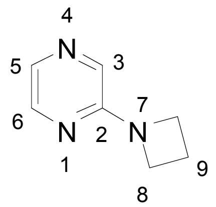

The title compound was synthesised following general procedure method A from 2-chloropyrazine (156 μL, 1.75 mmol), azetidine (118 μL, 1.75 mmol) and potassium fluoride (203 mg, 3.50 mmol) in water (1 mL) at 100 °C for 17 h to give the *title compound* as a colourless solid (114 mg, 48%); mp 64-66 °C; (Found; MH^+, 136.0861 $C_7H_{10}N_3^+$ requires 136.0869); ν_{max} (ATR)/cm^{-1} 2937, 2870, 1581, 1519, 1488, 1438; δ_H (400 MHz; $CDCl_3$) 8.03 (1H, dd, *J* 2.8, 1.5, 5-H), 7.84 (1H, d, *J* 2.8, 6-H), 7.78 (1H, d, *J* 1.5, 3-H), 4.15 (4H, t, *J* 7.6, 8-H), 2.49 (2H, quintet, *J* 7.6, 9-H); δ_C (75 MHz; $CDCl_3$) 156.0 (C), 142.0 (CH), 132.6 (CH), 130.2 (CH), 50.7 (CH_2), 17.2 (CH_2).

2-(Pyrrolidin-1-yl)pyrazine 8

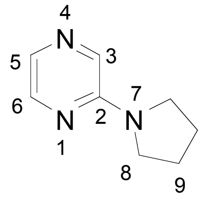

The title compound was synthesised following general procedure method A from 2-chloropyrazine (156 μL, 1.75 mmol), pyrrolidine (144 μL, 1.75 mmol) and potassium fluoride (203 mg, 3.50 mmol) in water (1 mL) at 100 °C for 17 h to give the *title compound* as a colourless solid (197 mg, 76%); mp 58-58 °C; (Found; MH^+, 150.1016. $C_8H_{12}N_3^+$ requires 150.1026); ν_{max} (ATR)/cm^{-1} 2963, 2870, 1577, 1518, 1488, 1462; δ_H (400 MHz; $CDCl_3$) 7.99 (1H, dd, *J* 2.2, 1.4, 5-H), 7.85 (1H, bs, 3-H), 7.74 (1H, d, *J* 2.2, 6-H), 3.46 (4H, dt, *J* 6.6, 1.9, 8-H), 2.02 (4H, dt, *J* 6.6, 1.9, 9-H); δ_C (75 MHz; $CDCl_3$) 153.0 (C), 142.0 (CH), 131.2 (CH), 130.8 (CH), 46.5 (CH_2), 25.4 (CH_2).

1-(Pyrazin-2-yl)azepane 9

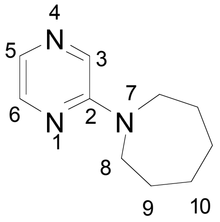

The title compound synthesised following general procedure method A from 2-chloropyrazine (156 μL, 1.75 mmol), azepane (210 μL, 1.75 mmol) and potassium fluoride (203 mg, 3.50 mmol) in water (1 mL) at 100 °C for 17 h to give the *title compound* as a yellow oil (161 mg, 52%); (Found; MH^+, 78.1340. $C_{10}H_{16}N_3^+$ requires 178.1339); ν_{max} (ATR)/cm^{-1} 2932, 2857, 1578, 1519, 1496, 1467; δ_H (400 MHz; $CDCl_3$) 8.03 (1H, dd, *J* 2.6, 1.5, 5-H), 8.02 (1H, d, *J* 1.5 , 3-H), 7.76 (1H, d, *J* 2.6, 6-H), 3.66 (4H, t, *J* 5.9, CH_2), 1.86-1.80 (4H, m, CH_2), 1.61-1.58 (4H, m, CH_2); δ_C (75 MHz; $CDCl_3$) 154.1 (C), 141.9 (CH), 131.0 (CH), 129.8 (CH), 47.2 (CH_2), 27.6 (CH_2), 27.1 (CH_2).

2-(4-Phenylpiperazin-1-yl)pyrazine 10

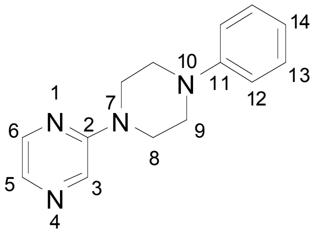

The title compound was synthesised following general procedure method A from 2-chloropyrazine (153 μL, 1.75 mmol), 1-phenylpiperazine (276 μL, 1.75 mmol) and potassium fluoride (203 mg, 3.50 mmol) in water (1 mL) at 100 °C for 17 h to give the *title compound* as a yellow solid (342 mg, 81%); yield based on NMR using 1,4-dioxan as internal standard; mp 112-114 °C (lit.,[1] mp 113-115 °C); (Found; MH^+, 241.1442. $C_{14}H_{17}N_4^+$ requires 241.1448); ν_{max}/cm^{-1} (ATR) 3021, 2847, 1597, 1574, 1519, 1483; δ_H (400 MHz; $CDCl_3$) 8.23 (1 H, d, *J*

1.5, 3-H), 8.12 (1 H, dd, *J* 2.7, 1.5, 5-H), 7.91 (1 H, d, *J* 2.7, 6-H), 7.35-7.28 (2 H, m, ArH), 7.02-6.94 (3 H, m, ArH), 3.79 (4 H, t, *J* 5.1, CH_2) 3.34 (4 H, t, *J* 5.1, CH_2); δ_C (75 MHz; $CDCl_3$) 155.0 (C), 151.1 (C), 141.8 (CH), 133.3 (CH), 131.2 (CH), 129.3 (CH), 120.4 (CH), 116.5 (CH), 49.1 (CH_2), 44.5 (CH_2).

_N_-Cyclohexylpyrimidin-2-amine 11

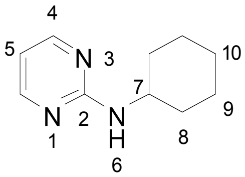

The title compound was synthesised following general procedure method A from 2-chloropyrimidine (200 mg, 1.75 mmol), cyclohexylamine (201 μL, 1.75 mmol), potassium fluoride (203 mg, 3.50 mmol) in water (1 mL) at 100 °C for 17 h to give the *title compound* as a cream solid (239 mg, 77%); mp 87-89 °C; (lit.,[3] mp 87-89 °C); (Found; MH^+, 178.1342. $C_{10}H_{16}N_3^+$ requires 178.1339); ν_{max}/cm^{-1} (ATR) 3261, 2922, 2850, 1585, 1563, 1523; δ_H (400 MHz; $CDCl_3$) 8.27 (2 H, d, *J* 4.7, 4-H), 6.50 (1 H, t, *J* 4.7, 5-H), 5.03 (1 H, bs, NH), 3.84 (1 H, tdt, *J* 10.1, 7.7, 3.8, 7-H), 2.08-2.04 (2 H, m, CH_2), 1.80-1.73 (2 H, m, CH_2), 1.69-1.63 (1H, m, CH_2), 1.49-1.43 (2H, m, CH_2), 1.31-1.18 (3H, m, CH_2); δ_C (75 MHz; $CDCl_3$) 161.8 (2C), 158.0 (CH), 110.1 (CH), 49.6 (CH), 33.2 (CH_2), 25.8 (CH_2), 24.9 (CH_2).

_N_-(4-Methoxybenzyl)pyrazin-2-amine 12

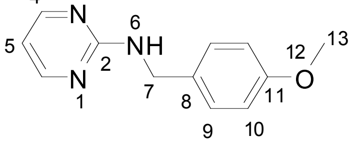

The title compound was synthesised following general procedure method A from 2-chloropyrimidine (200 mg, 1.75 mmol), 4-methoxybenzylamine (229 μL, 1.75 mmol) and potassium fluoride (203 mg, 3.50 mmol) in water (1 mL) at 100 °C for 17 h to give the *title compound* as a yellow solid (321 mg, 85%); mp 100-102 °C; (lit.,[3] mp 101-103 °C); (Found; MH^+, 216.1133. $C_{12}H_{14}N_3O^+$ requires 216.1131); ν_{max}/cm^{-1} (ATR) 3229, 2963, 1584, 1526, 1509, 1468; δ_H (400 MHz; $CDCl_3$) 8.31 (2H, d, *J* 4.7, 4-H), 7.30 (2H, d, *J* 8.8, ArH), 6.89 (2H, d, *J* 8.8, ArH), 6.57 (1H, t, *J* 4.7, 5-H), 5.44 (1H, bs, NH), 4.59 (2H, d, *J* 5.8, 7-H), 3.86 (3H, s, CH_3); δ_C (75 MHz; $CDCl_3$) 162.3 (C), 158.9 (C), 158.0 (CH), 131.1 (C), 128.9 (CH), 114.0 (CH), 110.7 (CH), 55.3 (CH_3), 45.0 (CH_2).

_N_-(2-(1_H_-Indol-3-yl)ethyl)pyrimidin-2-amine 13

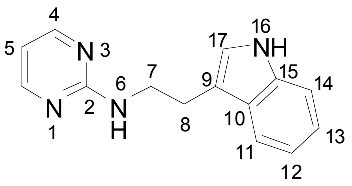

The title compound was synthesised following general procedure method A from 2-chloropyrimidine (200 mg, 1.75 mmol), tryptamine (280 mg, 1.75 mmol), potassium fluoride (203 mg, 3.50 mmol) in water (1 mL) at 100 °C for 17 h to give the *title compound* as a yellow solid (352 mg, 85%); mp 158-160 °C; (Found; MH^+, 239.1293. $C_{14}H_{15}N_4^+$ requires 239.1291); ν_{max}/cm^{-1} (ATR) 3172, 2925, 1594, 1541, 1452, 1417; δ_H (400 MHz; $CDCl_3$) 8.31 (2 H, d, *J* 4.7, 4-H), 8.06 (1 H, bs, NH), 7.68 (1 H, d, *J* 8.1, ArH), 7.40 (1 H, d, *J* 7.2, ArH), 7.23 (1 H, t, *J* 8.1, ArH), 7.16 (1 H, t, *J* 7.2, ArH), 7.10 (1 H, d, *J* 2.2, 17-H), 6.55 (1 H, t, *J* 4.7, 5-H), 5.29 (1 H, bs, NH), 3.79 (2 H, q, *J* 6.7, CH_2), 3.12 (2 H, q, *J* 6.7, CH_2); δ_C (75 MHz; $CDCl_3$) 162.3 (C), 158.1 (CH), 136.5 (C), 127.4 (C), 122.1 (CH), 119.4 (CH), 118.9 (CH), 113.2 (C), 111.2 (CH), 110.5 (CH), 41.4 (CH_2), 25.3 (CH_2).

***N*-(Fur-2-ylmethyl)pyrimidin-2-amine 14**

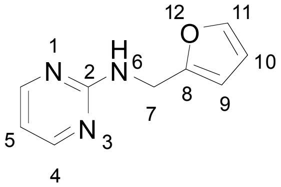

The title compound was synthesised following general procedure method A from 2-chloropyrimidine (200 mg, 1.75 mmol), furfurylamine (155 μL, 1.75 mmol) and potassium fluoride (203 mg, 1.75 mmol) in water (1 mL) at 100 °C for 17 h to give a mixture of starting material and the *title compound* as a brown solid (247 mg, 81%); mp 76-78 °C; (Found; MH^+, 176.0819. $C_9H_{10}N_3O^+$ requires 176.0818); ν_{max}/cm^{-1} (ATR) 3241, 2964, 1578, 1541, 1458, 1411;); δ_H (400 MHz; $CDCl_3$); 8.31 (2H, d, *J* 4.8, 4-H), 7.38 (1H, dd, *J* 1.8, 0.9, ArH), 6.58 (1H, t, *J* 4.8, 5-H), 6.34 (1H, dd, *J* 2.4, 1.8, ArH), 6.27 (1H, dd, *J* 2.4, 0.9, ArH), 5.66 (1H, bs, NH), 4.65 (1H, d, *J* 5.5, 7-H); δ_C (75 MHz; $CDCl_3$); 162.0 (C), 158.0 (CH), 152.4 (C), 142.0 (CH), 111.1 (CH), 110.3 (CH), 106.9 (CH), 38.6 (CH_2).

(*R*)-*N*-(1-Phenylethyl)pyrimidin-2-amine 15

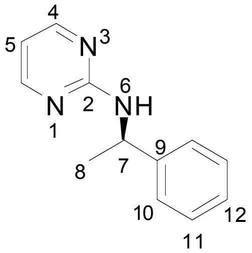

The title compound was synthesised following general procedure method A from 2-chloropyrimidine (200 mg, 1.75 mmol), (R)-1-phenylethylamine (212 mg, 1.75 mmol) and ptotassium fluoride (203 mg, 1.75 mmol) in water (1 mL) at 100 °C for 17 h to give the *title compound* as a yellow oil (272 mg, 78 %); (Found; MH^+, 200.1185. $C_{12}H_{14}N_3^+$ requires 200.1182); ν_{max}/cm^{-1} ($CHCl_3$) 3484, 2980, 1587, 1565, 1513, 1449; δ_H (400 MHz; $CDCl_3$) 8.21 (2H, d, *J* 4.8, 4-H), 7.42 (2H, d, *J* 7.1, ArH), 7.34 (2H, t, *J* 7.2, ArH), 7.28-7.24 (1H, m,

ArH), 6.48 (1H, t, *J* 4.8, 5-H), 6.34 (1H, d, *J* 6.9, NH), 5.25 (1H, quintet, *J* 6.9, 7-H), 1.59 (3H, d, *J* 6.9, 8-H); δ_C (75 MHz; $CDCl_3$) 161.8 (C), 158.0 (CH), 144.6 (C), 128.5 (CH), 126.9 (CH), 126.0 (CH), 110.5 (CH), 50.4 (CH), 23.0 (CH_3).

_N_-Benzyl-_N_-ethylpyrimidin-2-amine 16

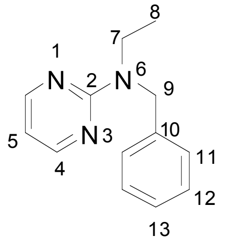

The title compound was synthesised following general procedure method A from 2-chloropyrimidine (200 mg, 1.75 mmol), *N*-ethylbenzylamine (260 μL, 1.75 mmol) and potassium fluoride (203 mg, 1.75 mmol) in water (1 mL) at 100 °C for 17 h to give the *title compound* as an orange oil (297 mg, 80%); (Found; MH^+, 214.1363. $C_{13}H_{16}N_3^+$ requires 214.1339); ν_{max}/cm^{-1} ($CDCl_3$) 3155, 2979, 1512, 1386, 907, 646; δ_H (400 MHz; $CDCl_3$) 8.68 (2 H, d, *J* 4.6, 4-H), 7.36-7.26 (5H, m, ArH), 6.50 (1 H, t, *J* 4.6, 5-H), 4.92 (2 H, s, 9-H), 3.66 (2 H, q, *J* 7.0, 7-H), 1.18 (3 H, t, *J* 7.0, 8-H) δ_C (75 MHz; $CDCl_3$) 161.7 (C), 157.8 (CH), 138.9 (C), 128.4 (CH), 127.3 (CH), 126.9 (CH), 109.4 (CH), 49.7 (CH_2), 41.6 (CH_2), 12.3 (CH_3). Data recorded matches literature.[4]

2-(Azetidin-1-yl)pyrimidine 17

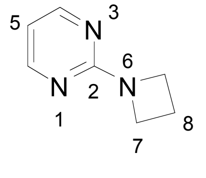

The title compound was synthesised following general procedure method A from 2-chloropyrimidine (200 mg, 1.75 mmol), azetidine (118 μL, 1.75 mmol) and potassium

fluoride (203 mg, 3.50 mmol) in water (1 mL) at 100 °C for 17 h to give the *title compound* as a yellow oil (123 mg, 52%); (Found; MH^+, 136.0875 $C_7H_{10}N_3^+$ requires 136.0869); ν_{max} (ATR)/cm^{-1} 2975, 2881, 1583, 1550, 1518, 1481; δ_H (400 MHz; $CDCl_3$) 8.32 (2H, d, *J* 4.8, 4-H), 6.51 (1H, t, *J* 4.8, 5-H), 4.18 (4H, t, *J* 7.6, 7-H), 2.40 (2H, quintet, *J* 7.6, 8-H), 2.49 (2H, quintet, *J* 7.6, 9-H); δ_C (75 MHz; $CDCl_3$) 162.6 (C), 157.9 (CH), 109.8 (CH), 50.0 (CH_2), 16.3 (CH_2).

2-(Pyrrolidin-1-yl)pyrimidine 18

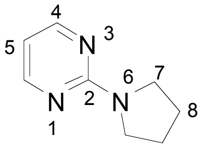

The title compound was synthesised following general procedure method A from 2-chloropyrimidine (200 mg, 1.75 mmol), azetidine (118 μL, 1.75 mmol) and potassium fluoride (203 mg, 3.50 mmol) in water (1 mL) at 100 °C for 17 h to give the *title compound* as a yellow oil (199 mg, 76%); (Found; MH^+, 150.1016. $C_8H_{12}N_3^+$ requires 150.1026); ν_{max} (ATR)/cm^{-1} 2975, 2873, 1586, 1547, 1518, 1486; δ_H (400 MHz; $CDCl_3$) 8.32 (2H, d, *J* 4.7, 4-H), 6.45 (1H, t, *J* 4.7, 5-H), 3.58 (4H, m, 7-H), 2.01 (4H, m, 8-H); δ_C (75 MHz; $CDCl_3$) 160.3 (C), 157.7 (CH), 108.8 (CH), 46.5 (CH_2), 25.5 (CH_2).

1-(Pyrimidin-2-yl)azepane 19

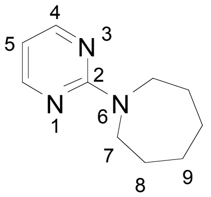

The title compound was synthesised following general procedure method A from 2-chloropyrimidine (200 mg, 1.75 mmol), azepane (197 μL, 1.75 mmol) and potassium fluoride

(203 mg, 3.50 mmol) in water (1 mL) at 100 °C for 17 h to give the *title compound* as a yellow oil (201 mg, 69 %); (Found; MH^+, 178.1344. $C_{10}H_{16}N_3^+$ requires 178.1339); ν_{max}/cm^{-1} ($CHCl_3$) 3666, 3176, 2933, 1591, 1500, 1434; δ_H (400 MHz; $CDCl_3$) 8.31 (2H, d, *J* 4.7, 4-H), 6.43 (1H, t, *J* 4.7, 5-H), 3.76 (4H, t, *J* 5.9, 7-H), 1.83-1.77 (4H, m, 8-H), 1.57 (4H, quintet, *J* 3.5, 9-H); δ_C (75 MHz; $CDCl_3$) 161.5 (C), 157.6 (CH), 108.7 (CH), 47.0 (CH_2), 27.9(CH_2), 27.3 (CH_2).

2-(4-Phenylpiperazin-1-yl)pyrimidine 20

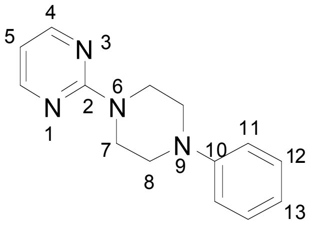

The title compound was synthesised following general procedure method A from 2-chloropyrimidine (200 mg, 1.75 mmol), 1-phenylpiperazine (276 μL, 1.75 mmol), potassium fluoride (203 mg, 3.50 mmol) in water (1 mL) at 100 °C for 17 h to give the *title compound* as a yellow solid (352 mg, 93%); (Found; MH^+, 241.1448. $C_{14}H_{17}N_4^+$ requires 241.1448); ν_{max}/cm^{-1} (ATR) 2833, 1581, 1543, 1494, 1439, 1360; δ_H (400 MHz; $CDCl_3$) 8.37 (2 H, d, *J* 4.7, 4-H), 7.32 (2 H, dt, *J* 8.8, 1.0, 12-H), 7.01 (2 H, dd, *J* 8.8, 1.0, 11-H), 6.92 (1 H, dt, *J* 8.8, 1.0, 13-H), 6.55 (1 H, t, *J* 4.7, 5-H), 4.01 (4 H, t, *J* 5.1, CH_2), 3.28 (4 H, t, *J* 5.1, CH_2); δ_C (75 MHz; $CDCl_3$) 161.7 (C), 157.8 (CH), 151.4 (C), 129.2 (CH), 120.2 (CH), 116.5 (CH), 110.1 (CH), 49.4 (CH_2), 43.7 (CH_2).

***N*-(4-Methoxyphenyl)pyrimidin-2-amine 21**

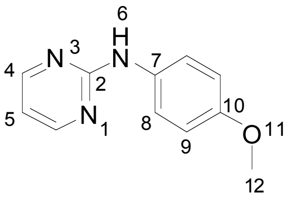

The title compound was synthesised following general procedure method A from 2-chloropyrimidine (200 mg, 1.75 mmol), *p*-anisidine (217 mg, 1.75 mmol), potassium fluoride (203 mg, 3.50 mmol) in water (1 mL) at 100 °C for 17 h to give the title compound as off-white solid (307 mg, 86%); mp 225-227 °C (lit.,[3] mp 224-226 °C); (Found; MH^+, 202.0989. $C_{11}H_{12}N_3O^+$ requires 202.0975); ν_{max}/cm^{-1} (ATR) 3254, 3010, 1609, 1576, 1530, 1507; δ_H (400 MHz; $CDCl_3$) 8.41 (2 H, d, *J* 4.7, 4-H), 7.50 (2 H, d, *J* 8.8, ArH), 6.98 (1 H, s, NH), 6.94 (2 H, d, *J* 8.8, ArH), 6.69 (1 H, t, *J* 4.7, 5-H) 3.84 (3 H, s, CH_3); δ_C (75 MHz; $CDCl_3$) 160.7 (C), 158.1 (CH), 155.8 (C), 132.4 (C), 122.3 (CH), 114.3 (CH), 112.0 (CH), 55.6 (CH_3).

2-(1*H*-Imidazol-1-yl)pyrimidine 22

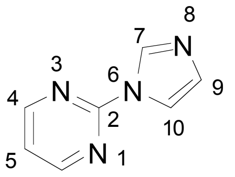

The title compound was synthesised following general procedure method A from 2-chloropyrimidine (200 mg, 1.75 mmol), imidazole (119 mg, 1.75 mmol) and potassium fluoride (203 mg, 1.75 mmol) in water (1 mL) at 100 °C for 17 h to give the *title compound* as a yellow solid (158 mg, 62%); mp 120-122 °C (lit.,[5] mp 124 °C); (Found; MH^+, 147.0660. $C_7H_7N_4^+$ requires 147.0665); ν_{max}/cm^{-1} (ATR) 3141, 2994, 1567, 1473, 1440, 1375; δ_H (400 MHz; $CDCl_3$) 8.72 (2 H, d, *J* 4.8, 4-H), 8.65 (1 H, s, ArH), 7.92 (1 H, t, *J* 1.4, ArH), 7.24 (1 H, t, *J* 4.8, 5-H), 7.20 (1 H, t, *J* 1.4, ArH); δ_C (75 MHz; $CDCl_3$) 158.7 (CH), 156.3 (CH), 136.2 (CH), 130.7 (CH), 118.9 (CH), 116.5 (CH).

6-(Pyrimidin-2-yl)-1*H*-benzimidazole 23

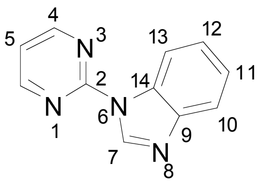

The title compound was synthesised following general procedure method A from 2-chloropyrimidine (200 mg, 1.75 mmol), benzimidazole (207 mg, 1.75 mmol) and potassium fluoride (203 mg, 3.50 mmol) in water (1 mL) at 100 °C for 17 h to give the *title compound* as a white solid (285 mg, 83%); mp 146-148 °C (lit.,[6] mp 149-150 °C); (Found; MH^+, 197.0841. $C_{11}H_9N_4^+$ requires 197.0822); ν_{max}/cm^{-1} ($CHCl_3$) 3138, 2987, 1575, 1501, 1479, 1458; δ_H (400 MHz; $CDCl_3$) 9.11 (1H, s, 7-H), 8.77 (1H, d, *J* 4.8, 4-H), 8.63-8.60 (1H, m, Ar-H), 7.88-7.85 (1H, m, Ar-H), 7.46-7.42 (1H, m, Ar-H), 7.41-7.37 (1H, m, Ar-H), 7.31 (1H, t, *J* 4.8, 5-H); δ_C (75 MHz; $CDCl_3$) 158.5 (CH), 156.4 (C), 144.8 (C), 141.8 (CH), 131.8 (C), 124.6 (CH), 123.8 (CH), 120.4 (CH), 118.1 (CH), 115.6 (CH).

4-(Pyrrolidin-1-yl)pyrimidine-2,6-diamine 24

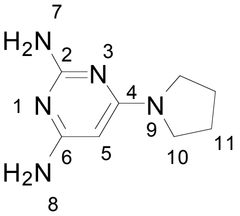

The title compound was synthesised following general procedure method A from 2,6-diamino-4-chloropyrimidine (253 mg, 1.75 mmol), pyrrolidine (144 μL, 1.75 mmol) and potassium fluoride (203 mg, 3.50 mmol) in water (1 mL) at 100 °C for 17 h to give the *title compound* as a colourless solid (142 mg, 45%); mp 176-178 °C; (Found; MH^+, 180.1249. $C_8H_{14}N_5^+$ requires 180.1244); ν_{max}/cm^{-1} (ATR) 3497, 2976, 1652, 1550, 1496, 1427; δ_H (400

MHz; $CDCl_3$) 4.96 (1H, s, 5-H), 4.50 (2H, s, NH), 4.26 (2H, s, NH), 3.40 (4H, bs, 10-H), 1.97-1.94 (4H, m, 11-H); δ_C (75 MHz; $CDCl_3$); 163.6 (C), 162.6 (C), 162.3 (C), 75.3 (CH), 46.2 (CH_2), 25.3 (CH_2).

4-Morpholinopyrimidine-2,6-diamine 25

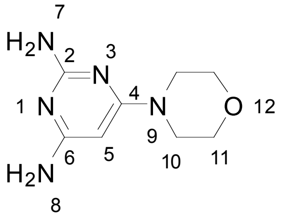

The title compound was synthesised following general procedure method A from 2,6-diamino-4-chloropyrimidine (253 mg, 1.75 mmol), morpholine (153 μL, 1.75 mmol) and potassium fluoride (203 mg, 3.50 mmol) in water (1 mL) at 100 °C for 17 h to give the *title compound* as a colourless solid (158 mg, 49%); mp 177-179 °C; (Found; MH^+, 196.1201. $C_8H_{14}N_5O^+$ requires 196.1193); ν_{max}/cm^{-1} (ATR) 3349, 3175, 1655, 1611, 1560, 1425; δ_H (400 MHz; $CDCl_3$) 5.14 (1H, s, 5-H), 4.52 (2H, bs, NH), 4.35 (2H, bs, NH), 3.76 (4H, t, *J* 4.8, CH_2), 3.50 (2H, t, *J* 4.8, CH_2); δ_C (75 MHz; $CDCl_3$) 164.8 (C), 164.7 (C), 162.6 (C), 75.3 (CH), 66.6 (CH_2), 44.5 (CH_2).

4-(4-Phenylpiperazin-1-yl)pyrimidine-2,6-diamine 26

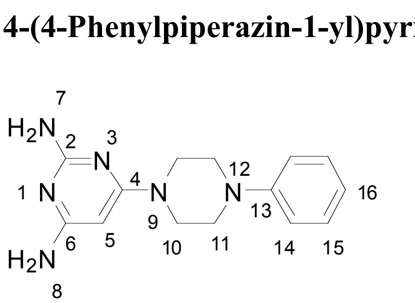

The title compound was synthesised following general procedure method A from 2,6-diamino-4-chloropyrimidine (253 mg, 1.75 mmol), 1-phenylpiperazine (153 μL, 1.75 mmol) and potassium fluoride (203 mg, 3.50 mmol) in water (1 mL) at 100 °C for 17 h to give the

title compound as a yellow solid (379 mg, 80%); mp 209-211 °C; (Found; MH^+, 271.1660. $C_{14}H_{19}N_6^+$ requires 271.1666); ν_{max}/cm^{-1} (ATR) 3412, 2824, 1557, 1489, 1439, 1415; δ_H (400 MHz; $CDCl_3$) 7.31 (2H, dd, *J* 8.8, 1.0, ArH), 6.98 (2H, dd, *J* 8.8, 1.0, ArH), 6.91 (1H, dt, *J* 8.8, 1.0, ArH), 5.21 (1H, s, 5-H), 4.54 (2H, bs, NH), 4.36 (2H, bs, NH), 3.70 (4H, t, *J* 5.2, CH_2), 3.25 (4H, t, *J* 5.2, CH_2); δ_C (75 MHz; $CDCl_3$) 164.6 (C), 164.5 (C), 162.6 (C), 151.2 (C), 129.2 (CH), 120.2 (CH), 116.4 (CH), 75.4 (CH), 49.1 (CH_2), 44.1 (CH_2).

_N_-Cyclohexylquinazolin-4-amine 27

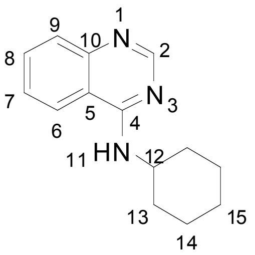

The title compound was synthesised following general procedure method A from 4-chloroquinazoline (288 mg, 1.75 mmol), cyclohexylamine (201 μL, 1.75 mmol) and potassium fluoride (203 mg, 3.50 mmol) in water (1 mL) at 100 °C for 17 h to give the *title compound* as a yellow solid (309 mg, 78%); mp 136-138 °C (lit.,[7] mp 138-140 °C); (Found; MH^+, 228.1500. $C_{14}H_{18}N_3^+$ requires 228.1495); ν_{max}/cm^{-1} ($CHCl_3$)3453, 2937, 2857, 1684, 1617, 1580, 1528; δ_H (400 MHz; $CDCl_3$) 8.67 (1H, s, 2-H), 7.86 (1H, d, *J* 7.9, ArH), 7.76-7.70 (2H, m, ArH), 7.48 (1H, dt, *J* 7.9, 1.1, ArH), 5.65 (1H, bs, NH), 4.34-4.24 (1H, m, 12-H), 2.20-2.16 (2H, m, CH_2), 1.86-1.81 (2H, m, CH_2), 1.76-1.70 (1H, m, CH_2), 1.58-1.47 (2H, m, CH_2), 1.39-1.24 (3H, m, CH_2); δ_C (75 MHz; $CDCl_3$) 158.6 (C), 155.4 (CH), 149.2 (C), 132.5 (CH), 128.4 (CH), 125.9 (CH), 120.4 (CH), 114.8 (C), 49.7 (CH), 33.1 (CH_2), 25.7 (CH_2), 25.0 (CH_2).

_N_-(_p_-Methoxybenzyl)quinazolin-4-amine 28

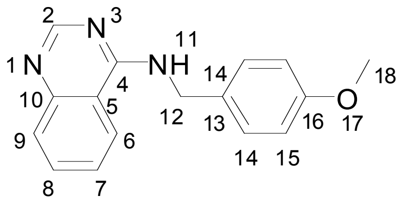

The title compound was synthesised following general procedure method A from 4-chloroquinazoline (288 mg, 1.75 mmol), 4-methoxybenzyl amine (229 μL, 1.75 mmol) and potassium fluoride (203 mg, 3.50 mmol) in water (1 mL) at 100 °C for 17 h to give the *title compound* as a yellow solid (331 mg, 71%); mp 157-159 °C; (Found; MH^+, 266.0611. $C_{16}H_{16}N_3O^+$ requires 266.1288); ν_{max}/cm^{-1} ($CHCl_3$) 3459, 2963, 2838, 1616, 1578, 1534; δ_H (400 MHz; $CDCl_3$) 8.73 (1H, s, 2-H), 7.87 (1H, d, *J* 8.8, ArH), 7.77-7.71 (2H, m, ArH), 7.46 (2H, dt, *J* 7.0, 1.3, ArH), 7.36 (1H, d, *J* 8.7, ArH), 6.92 (2H, d, *J* 8.7, ArH), 6.03 (1H, bs, NH), 4.81 (2H, d, *J* 5.3, CH_2), 3.81 (3H, s, CH_3); δ_C (75 MHz; $CDCl_3$) 159.3 (C), 159.2 (C), 155.4 (CH), 149.5 (C), 132.6 (CH), 130.1 (C), 129.5 (CH), 128.6 (CH), 126.0 (CH), 120.5 (CH), 114.9 (C), 114.2 (CH), 55.3 (CH_2), 44.9 (CH_3). ^1H NMR data recorded matches literature.[8]

***N*-(Fur-2-ylmethyl)quinazolin-4-amine 29**

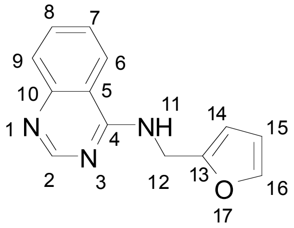

The title compound was synthesised following general procedure method A from 4-chloroquinazoline (288 mg, 1.75 mmol), furfurylamine (155 μL, 1.75 mmol) and potassium fluoride (203 mg, 3.50 mmol) in water (1 mL) at 100 °C for 17 h to give the *title compound* as a beige solid (281 mg, 71%); mp 170-172 °C; (Found; MH^+, 226.0978. $C_{13}H_{12}N_3O^+$ requires 226.0975); ν_{max}/cm^{-1} ($CHCl_3$) 3693, 3465, 2975, 1601, 1578, 1532; δ_H (400 MHz; $CDCl_3$)

8.73 (1H, s, 2-H), 7.87 (1H, d, *J* 8.2, ArH), 7.78-7.73 (2H, m, ArH), 7.47 (1H, dt, *J* 8.2, 1.1, ArH), 7.41 (1H, t, *J* 1.1, ArH), 6.37 (2H, d, *J* 1.5, ArH), 6.23 (1H, bs, NH), 4.89 (2H, d, *J* 5.3, CH_2); δ_C (75 MHz; $CDCl_3$) 159.1 (C), 155.2 (CH), 151.1 (C), 149.3 (C), 142.4 (CH), 132.8 (CH), 128.4 (CH), 126.2 (CH), 120.7 (CH), 114.9 (C), 110.6 (CH), 108.1 (CH), 38.2 (CH_2).

4-(Pyrrolidin-1-yl)quinazoline 30

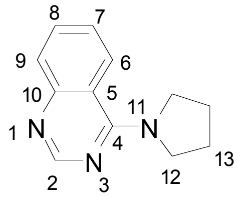

The title compound was synthesised following general procedure method A from 4-chloroquinazoline (288 mg, 1.75 mmol), pyrrolidine (144 μL, 1.75 mmol) and potassium fluoride (203 mg, 3.50 mmol) in water (1 mL) at 100 °C for 17 h to give the *title compound* as a brown solid (203 mg, 97%); mp 35-37 °C (lit.,[7] mp 39-42 °C); (Found; MH^+, 200.1189. $C_{12}H_{14}N_3^+$ requires 200.1182); δ_H (400 MHz; $CDCl_3$) 8.61 (1H, s, 2-H), 8.17 (1H, dd, *J* 8.4, 1.1, ArH), 7.84 (2H, dd, *J* 8.4, 1.1, ArH), 7.70 (1H, dt, *J* 8.4, 1.4, ArH), 7.39 (1H, dt, *J* 8.4, 1.4, ArH), 3.95 (4H, t, *J* 6.7, 12-H), 2.09-2.05 (4H, m, 13-H); δ_C (75 MHz; $CDCl_3$) 159.8 (C), 154.4 (CH), 151.3 (C), 132.0 (CH), 128.0 (CH), 125.3 (CH), 124.4 (CH), 116.5 (C), 51.0 (CH_2), 25.7 (CH_2).

4-(Quinazolin-4-yl)morpholine 31

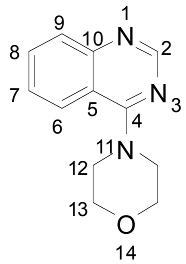

The title compound was synthesised following general procedure method A from 4-chloroquinazoline (288 mg, 1.75 mmol), morpholine (153 µL, 1.75 mmol) and potassium fluoride (203 mg, 3.50 mmol) in water (1 mL) at 100 °C for 17 h to give the *title compound* as a yellow solid (301 mg, 80%); mp 92-94 °C (lit.,[7] mp 87-90 °C; lit.,[9] mp 95-96 °C); (Found; MH^+, 216.1135. $C_{12}H_{14}N_3O^+$ requires 216.1131); ν_{max}/cm^{-1} ($CHCl_3$) 2971, 2860, 1687, 1614, 1568, 1545; δ_H (400 MHz; $CDCl_3$) 8.69 (1H, s, 2-H), 7.85 (1H, d, *J* 8.4, ArH), 7.81 (1H, d, *J* 8.4, ArH), 7.69-7.65 (1H, m, ArH), 7.42-7.38 (1H, m, ArH), 3.83 (4H, t, *J* 4.9, CH_2), 3.71 (4H, t, *J* 4.9, CH_2); δ_C (75 MHz; $CDCl_3$) 164.6 (C), 153.9 (CH), 151.7 (C), 132.6 (CH), 128.6 (CH), 125.6 (CH), 124.6 (CH), 116.5 (C), 66.7 (CH_2), 50.2 (CH_2).

4-(4-Phenylpiperazin-1-yl)quinazoline 32

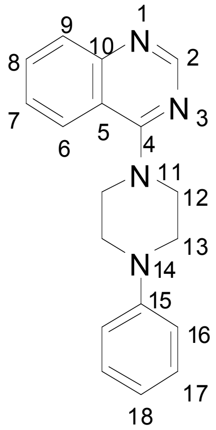

The title compound was synthesised following general procedure method A from 4-chloroquinazoline (277 mg, 1.75 mmol), 1-phenylpiperazine (276 µL, 1.75 mmol) and potassium fluoride (203 mg, 3.50 mmol) in water (1 mL) at 100 °C for 17 h to give the *title compound* as a yellow solid (418 mg, 82%); mp 89-90 °C (lit.,[10] oil); (Found; MH^+, 291.1593. $C_{18}H_{19}N_4^+$ requires 291.1604); ν_{max} ($CDCl_3$)/cm^{-1} 2950, 2844, 1599, 1569, 1547, 1492; δ_H (400 MHz; $CDCl_3$) 8.80 (1 H, s, 2-H), 7.96 (2 H, t, *J* 8.6, ArH), 7.79 (1 H, dt, *J* 7.0, 1.3, ArH), 7.52 (1 H, dt, *J* 7.0, 1.3, ArH), 7.34 (2 H, dd, *J* 8.6, 1.4, ArH), 7.02 (2 H, d, *J* 8.6,

ArH), 6.94 (1 H, t, J 7.0, ArH), 3.97 (4 H, t, J 5.1, CH_2), 3.44 (4 H, t, J 5.1, CH_2); δ_C (75 MHz; $CDCl_3$) 164.7 (C), 154.0 (CH), 151.7 (C), 151.0 (C), 132.6 (CH), 129.3 (CH), 128.7 (CH), 125.6 (CH), 124.9 (CH), 120.3 (CH), 116.7 (C), 116.3 (CH), 49.7 (CH_2), 49.2 (CH_2).

***N*-(*p*-Methoxyphenyl)quinazolin-4-amine 33**

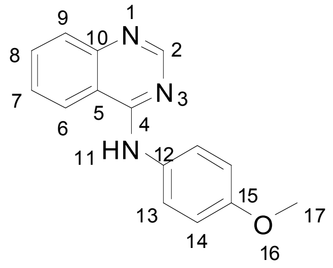

The title compound was synthesised following general procedure method A from 4-chloroquinazoline (288 mg, 1.75 mmol), *p*-anisidine (218 mg, 1.75 mmol) and potassium fluoride (203 mg, 3.50 mmol) in water (1 mL) at 100 °C for 17 h to give the *title compound* as a cream solid (378 mg, 86%); mp 166-168 (lit.,[7] mp 169-171 °C; (Found; MH^+, 252.1133. $C_{15}H_{14}N_3O^+$ requires 252.1131); ν_{max}/cm^{-1} ($CHCl_3$) 3452, 3005, 2838, 1688, 1618, 1599; δ_H (400 MHz; $CDCl_3$) 8.74 (1H, s, 2-H), 7.93 (2H, dd, J 8.6, 1.0, ArH), 7.81 (1H, dt, J 8.6, 1.0, ArH), 7.62-7.55 (3H, m, ArH), 6.99 (2H, d, ArH), 3.86 (3H, s, CH_3); δ_C (75 MHz; $CDCl_3$) 158.0 (C), 157.1 (C), 155.1 (CH), 149.8 (C), 132.9 (CH), 130.8 (C), 128.9 (CH), 126.5 (CH), 124.5 (CH), 120.4 (CH), 115.1 (C), 114.4 (CH), 55.6 (CH_3).

2-(Pyrrolidin-1-yl)pyridine 34

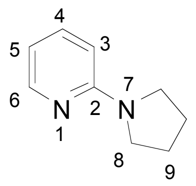

The title compound was synthesised following general procedure method A from 2-fluoropyridine (151 μL, 1.75 mmol), pyrrolidine (144 μL, 1.75 mmol) and potassium fluoride

(203 mg, 3.50 mmol) in water (1 mL) at 100 °C for 17 h to give the *title compound* as a yellow oil (139 mg, 54 %); (lit.,[11] oil); (Found; MH^+, 149.1067. $C_9H_{13}N_2^+$ requires 149.1073); ν_{max}/cm^{-1} ($CHCl_3$) 2973, 2856, 1630, 1600, 1555, 1502; δ_H (400 MHz; $CDCl_3$) 8.17 (1H, ddd, *J* 5.1, 1.9, 0.9, ArH), 7.43 (1H, ddd, *J* 8.8, 7.0, 1.9, ArH), 6.51 (1H, ddd, *J* 7.0, 5.1, 0.9, ArH), 6.35 (1H, d, *J* 8.8, ArH), 4.50 (1H, bs, NH), 3.48-3.44 (4H, m, 8-H), 2.03-2.00 (4H, m, 9-H); δ_C (75 MHz; $CDCl_3$) 157.3 (C), 148.2 (CH), 136.9 (CH), 111.0 (CH), 106.5 (CH), 46.6 (CH_2), 25.5 (CH_2).

4-(Pyridin-2-yl)morpholine 35

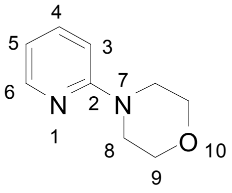

The title compound was synthesised following general procedure method A from 2-chloropyridine (167 μL, 1.75 mmol), morpholine (153 μL, 1.75 mmol) and potassium fluoride (203 mg, 3.50 mmol) in water (1 mL) at 100 °C for 17 h to give the title compound as a yellow semi-solid (27 mg, 9%); (Found; MH^+, 165.1040. $C_9H_{13}N_2O^+$ requires 165.1022); ν_{max} ($CDCl_3$)/cm^{-1} 3055, 1594, 1481, 1437, 1376, 1313; δ_H (400 MHz; $CDCl_3$) 8.24 (1 H, d, *J* 3.7, ArH), 7.53 (1 H, t, *J* 8.8, ArH), 6.69 (2 H, m, ArH), 3.86 (4 H, s, CH_2), 3.52 (4 H, s, CH_2); δ_C (75 MHz; $CDCl_3$) 159.6 (C), 148.0 (CH), 137.5 (CH), 113.8 (CH), 107.0 (CH), 66.8 (CH_2), 45.6 (CH_2). Data recorded matches literature.[12]

1-Phenyl-4-(pyridine-2-yl)piperazine 36

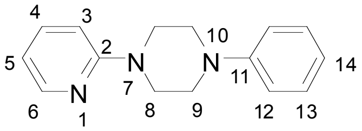

The title compound was synthesised following general procedure method A from 2-chloropyridine (167 μL, 1.75 mmol), 1-phenylpiperazine (276 μL, 1.75 mmol) and potassium fluoride (203 mg, 3.50 mmol) in water (1 mL) at 100 °C for 17 h to give the *title compound* as a white solid (88 mg, 21%); yield based on NMR using 1,4-dioxan as internal standard; mp 100-102 °C; (Found; MH^+, 240.1503. $C_{15}H_{18}N_3^+$ requires 240.1495); ν_{max}/cm^{-1} ($CHCl_3$) 3010, 2888, 2836, 1596, 1562, 1483; δ_H (400 MHz; $CDCl_3$) 8.25 (1H, dd, *J* 4.9, 2.0, ArH), 7.54 (1H, ddd, *J* 7.1, 2.0, 0.7, ArH), 7.32 (2H, dd, *J* 8.8, 1.0, ArH), 7.02 (2H, dd, *J* 8.8, 1.0, ArH), 6.92 (1H, tt, *J* 8.8, 1.0, ArH), 6.73 (1H, d, *J* 7.1, ArH), 5.25 (1H, ddd, *J* 4.9, 1.3, 0.7, ArH), 3.74 (4H, t, *J* 5.1, CH_2), 3.34 (4H, t, *J* 5.1, CH_2); δ_C (75 MHz; $CDCl_3$); 159.5 (C), 151.3 (C), 148.0 (CH), 137.6 (CH), 129.2 (CH), 120.1 (CH), 116.4 (CH), 113.6 (CH), 107.2 (CH), 49.2 (CH_2), 45.3 (CH_2).

_N_-Cyclohexyl-5-(trifluoromethyl)pyridine-2-amine 37

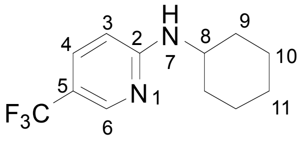

The title compound was synthesised following general procedure method A from 2- chloro, 5-trifluoromethyl pyridine (318 mg, 1.75 mmol), cyclohexylamine (201 μL, 1.75 mmol) and potassium fluoride (203 mg, 3.50 mmol) in water (1 mL) at 100 °C for 17 h to give the *title compound* as a brown solid (235 mg, 60%); mp 76-78 °C; (Found; MH^+, 245.1275. $C_{12}H_{16}N_2F_3^+$ requires 245.1260); ν_{max}/cm^{-1} ($CHCl_3$) 3417, 2935, 2857, 1615, 1575, 1531; δ_H (400 MHz; $CDCl_3$) 8.32 (1H, s, 6-H), 7.56 (1H, dd, *J* 8.9, 2.5, 4-H), 6.37 (1H, d, *J* 8.9, 3-H), 4.94 (1H, bs , NH), 3.61 (1H, m, 8-H), 2.06-2.02 (2H, m, CH_2), 1.80-1.75 (2H, m, CH_2), 1.70-1.63 (1H, m, CH_2), 1.47-1.36 (2H, m, CH_2), 1.30-1.19 (3H, m, CH_2); δ_C (75 MHz; $CDCl_3$);

159.7 (C), 146.2 (q, ${}^{3}J_{CF}$ 5.0, CH), 134.2 (q, ${}^{3}J_{CF}$ 3.0, CH), 124.7 (q, ${}^{1}J_{CF}$ 269, CF_3), 114.8 (q, ${}^{2}J_{CF}$ 32.9, C), 105.9 (CH), 50.2 (CH), 33.0 (CH_2), 25.6 (CH_2), 24.6 (CH_2).

***N*-(4-Methoxybenzyl)-5-trifluoromethyl)pyridine-2-amine 38**

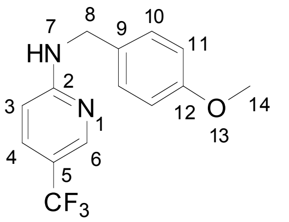

The title compound was synthesised following general procedure method A from 2- chloro-5-trifluoromethyl pyridine (318 mg, 1.75 mmol), 4-methoxybenzylamine (229 μL, 1.75 mmol) and potassium fluoride (203 mg, 3.50 mmol) in water (1 mL) at 100 °C for 17 h to give the *title compound* as a white solid (322 mg, 65%); mp 162-164 °C; (Found; MH^+, 283.1051. $C_{14}H_{14}N_2F_3O^+$ requires 283.1053); ν_{max}/cm^{-1} ($CHCl_3$) 3443, 2337, 1615, 1514, 1466, 1410; δ_H (400 MHz; $CDCl_3$) 8.35 (1H, s, 6-H), 7.59 (1H, dd, *J* 8.9, 2.5, 4-H), 7.29 (2H, d, *J* 8.8, ArH), 6.91 (2H, d, *J* 8.8, ArH), 6.41 (1H, d, *J* 8.9, 3-H), 5.26 (1H, bs, NH), 4.50 (2H, d, *J* 5.6, 8-H), 3.83 (3H, s, CH_3); δ_C (75 MHz; $CDCl_3$) 160.2 (C), 159.1 (C), 146.1 (CH), 134.5 (CH), 130.2 (C), 128.9 (CH), 128.3 (C), 114.2 (CH), 113.9 (C), 106.0 (CH), 55.3 (CH_3) 45.6 (CH_2).

***N*-(Furan-2-ylmethyl)-5-(trifluoromethyl)pyridine-2-amine 39**

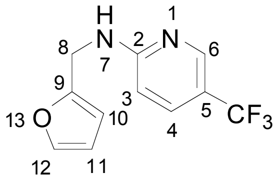

The title compound was synthesised following general procedure method A from 2- chloro-5-trifluoromethyl pyridine (318 mg, 1.75 mmol), furfurylamine (155 μL, 1.75 mmol) and potassium fluoride (203 mg, 3.50 mmol) in water (1 mL) at 100 °C for 17 h to give the *title compound* as a white solid (205 mg, 48%); mp 99-101 °C; (Found; MH^+, 243.0745.

$C_{11}H_{10}N_2F_3O^+$ requires 243.0740); ν_{max}/cm^{-1} ($CHCl_3$) 3445, 1616, 1578, 1522, 1486, 1411; δ_H (400 MHz; $CDCl_3$) 8.37 (1H, s, 6-H), 7.61 (1H, dd, *J* 8.8, 2.5, 4-H), 7.39 (1H, dd, *J* 1.9, 0.9, ArH), 6.49 (1H, d, *J* 8.8, 3-H), 6.36 (1H, dd, *J* 2.6, 1.9, ArH), 6.28 (1H, dd, *J* 2.6, 0.9, ArH), 5.31 (1H, s, NH), 4.59 (2H, d, *J* 5.7, 8-H); δ_C (75 MHz; $CDCl_3$) 159.8 (C), 151.6 (C), 146.0 (q, $^3J_{CF}$ 4.6, CH), 142.2 (CH), 134.4 (q, $^3J_{CF}$ 3.0, CH), 125.9 (CF_3), 115.6 (C), 110.5 (CH), 107.4 (CH), 106.6 (CH), 39.0 (CH_2).

2-(Pyrrolidin-1-yl)-5-(trifluoromethyl)pyridine 40

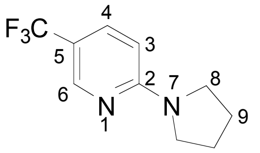

The title compound was synthesised following general procedure method A from 2-chloro-5-trifluoromethylpyridine (318 mg, 1.75 mmol), pyrrolidine (144 μL, 1.75 mmol) and potassium fluoride (203 mg, 3.50 mmol) in water (1 mL) at 100°C for 17 h to give the *title compound* as a cream solid (199 mg, 53%); mp 65-67 °C; (Found; MH^+, 217.0954. $C_{10}H_{12}N_2F_3^+$ requires 217.0947); ν_{max}/cm^{-1} ($CHCl_3$) 2977, 2871, 1613, 1558, 1523, 1486; δ_H (400 MHz; $CDCl_3$) 8.38 (1H, d, *J* 2.5, 6-H), 7.57 (1H, dd, *J* 8.9, 2.5, 4-H), 6.34 (1H, d, *J* 8.9, 3-H), 3.48 (4H, bs, 8-H), 2.04-2.01 (4H, m, 9-H); δ_C (75 MHz; $CDCl_3$) 158.4 (C), 146.0 (q, $^3J_{CF}$ 4.6, CH), 133.7 (q, $^3J_{CF}$ 3.1, CH), 124.6 (q, $^1J_{CF}$ 269.9, CF_3), 113.5 (q, $^2J_{CF}$ 32.9, C), 105.6 (CH), 46.8 (CH_2), 25.4 (CH_2).

2-(5-(Trifluoromethyl)pyridin-2-yl)morpholine 41

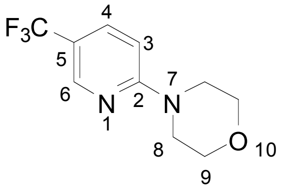

The title compound was synthesised following general procedure method A from 2-chloro-5-trifluoromethyl pyridine (318 mg, 1.75 mmol), morpholine (153 μL, 1.75 mmol) and potassium fluoride (203 mg, 3.50 mmol) in water (1 mL) at 100 °C for 17 h to give the *title compound* as a white solid (145 mg, 36%); mp 58-60 °C; (Found; MH^+, 233.0910. $C_{10}H_{12}N_2F_3O^+$ requires 233.0896); ν_{max}/cm^{-1} ($CHCl_3$) 2973, 2898, 2861, 1613, 1563, 1508; δ_H (400 MHz; $CDCl_3$) 8.43 (1H, s, 6-H), 7.67 (1H, d, *J* 8.9, 4-H), 6.65 (1H, d, *J* 8.9, 3-H), 3.83 (4H, m , CH_2), 3.63 (4H, m, CH_2); δ_C (75 MHz; $CDCl_3$); 160.6 (C), 145.7 (q, $^3J_{CF}$ 3.8, CH), 134.6 (q, $^3J_{CF}$ 3.9, CH), 124.3 (q, $^1J_{CF}$ 269.9, CF_3), 115.7 (q, $^2J_{CF}$ 33.0, C), 105.5 (CH), 66.5 (CH_2), 45.0 (CH_2).

1-Phenyl-4-(5-(trifluoromethyl)pyridine-2-yl)piperazine 42

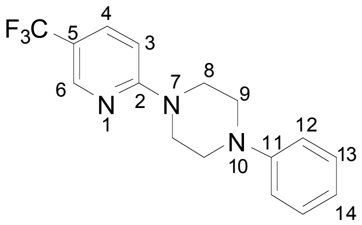

The title compound was synthesised following general procedure method A from 2-chloro-5-trifluoromethylpyridine (318 mg, 1.75 mmol), 1-phenylpiperazine (276 μL, 1.75 mmol) and potassium fluoride (203 mg, 3.50 mmol) in water (1 mL) at 100°C for 17 h to give the *title compound* as a white solid (456 mg, 85%); mp 119-121 °C; (Found; MH^+, 308.1360. $C_{16}H_{17}N_3F_3^+$ requires 308.1369); ν_{max}/cm^{-1} ($CHCl_3$) 3010, 2832, 1613, 1563, 1505, 1453; δ_H (400 MHz; $CDCl_3$) 8.45 (1H, bs, 6-H), 7.68 (1H, dd, *J* 8.9, 2.0, 4-H), 7.32 (2H, dd, *J* 8.9, 7.3, ArH), 7.00 (2H, dd, *J* 8.9, 1.0, ArH), 6.93 (1H, dt, *J* 7.3, 1.0, ArH), 6.72 (1H, d, *J* 8.9, 3-H), 3.84 (4H, t, *J* 5.1, CH_2), 3.33 (4H, t, *J* 5.1, CH_2); δ_C (75 MHz; $CDCl_3$) 160.3 (C), 151.0 (C), 145.8 (q, $^3J_{CF}$ 3.9, CH), 134.6 (q, $^3J_{CF}$ 3.0, CH), 129.3 (CH), 124.3 (q, $^1J_{CF}$ 270.0, CF_3), 120.0 (CH), 116.4 (CH), 115.3 (q, $^2J_{CF}$ 32.9, C), 105.7 (CH), 49.1 (CH_2), 44.7 (CH_2).

_N_-Cyclohexyl-5-nitropyridin-2-amine 43

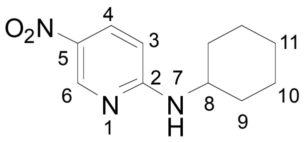

The title compound was synthesised following general procedure method A from 2-chloro,5-nitropyridine (277 mg, 1.75 mmol), cyclohexylamine (201 μL, 1.75 mmol) and potassium fluoride (203 mg, 3.50 mmol) in water (1 mL) at 100 °C for 17 h to give the *title compound* as a yellow solid (372 mg, 96%); mp 127-129 °C; (Found; MH^+, 222.1243. $C_{11}H_{16}N_3O_2^+$ requires 222.1237); ν_{max}/cm^{-1} ($CHCl_3$) 3431, 3408, 2937, 2857, 1603, 1580; δ_H (400 MHz; $CDCl_3$) 8.97 (1H, d, *J* 2.5, 6-H), 8.13 (1H, dd, *J* 8.9, 2.5, 4-H), 6.35 (1H, d, *J* 8.9, 3-H), 5.64 (1H, bs, NH), 3.71 (1H, bs, 8-H), 2.05-2.01 (2H, m, CH_2), 1.79-1.74 (2H, m, CH_2), 1.68-1.62 (1H, m, CH_2), 1.45-1.35 (2H, m, CH_2), 1.31-1.18 (3H, m, CH_2); δ_C (75 MHz; $CDCl_3$) 160.5 (C), 147.2 (CH), 135.2 (C), 132.8 (CH), 105.5 (CH), 50.7 (CH), 32.8 (CH_2), 25.6 (CH_2), 24.7 (CH_2).

_N_-(4-Methoxybenzyl)-5-nitropyridin-2-amine 44

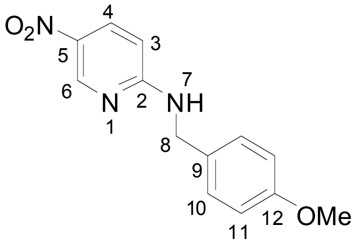

The title compound was synthesised following general procedure method A from 2-chloro-5-nitropyridine (277 mg, 1.75 mmol), 4-methoxybenzylamine (229 μL, 1.75 mmol) and potassium fluoride (203 mg, 3.50 mmol) in water (1 mL) at 100 °C for 17 h to give the *title compound* as an orange solid (314 mg, 70%); mp 138-140 °C; (Found; MH^+, 260.1031. $C_{13}H_{14}N_3O_3^+$ requires 260.1030); ν_{max}/cm^{-1} ($CHCl_3$) 3438, 3009, 2839, 1604, 1581, 1513; δ_H

(400 MHz; $CDCl_3$) 8.95 (1H, s, 6-H), 8.19 (1H, dd, *J* 9.2, 2.5, 4-H), 7.28 (2H, d, *J* 8.8, ArH), 6.92 (2H, d, *J* 8.8, ArH), 6.39 (1H, d, *J* 9.2, 3-H), 4.56 (2H, d, *J* 4.7, 8-H), 3.83 (3H, s, CH_3); δ_C (75 MHz; $CDCl_3$) 161.0 (C), 159.4 (C), 146.9 (CH), 136.0 (C), 133.1 (CH), 132.0 (C), 129.0 (CH), 114.3 (CH), 105.0 (CH), 55.3 (CH_3), 45.9 (CH_2).

_N_-(Furan-2-ylmethyl)-5-nitropyridin-2-amine 45

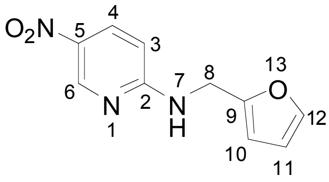

The title compound was synthesised following general procedure method A from 2-chloro,5-nitropyridine (277 mg, 1.75 mmol), furfurylamine (155 μL, 1.75 mmol) and potassium fluoride (203 mg, 3.50 mmol) in water (1 mL) at 100 °C for 17 h to give the *title compound* as a orange solid (315 mg, 73%); mp 108-110 °C; (Found; MH^+, 220.0724. $C_{10}H_{10}N_3O_3^+$ requires 220.0717); ν_{max}/cm^{-1} ($CHCl_3$) 3444, 3011, 2931, 1605, 1582, 1528; δ_H (400 MHz; $CDCl_3$) 9.05 (1H, d, *J* 2.7, 6-H), 8.22 (1H, dd, *J* 9.4, 2.7, 4-H), 7.41 (1H, dd, *J* 1.9, 0.7, ArH), 6.47 (1H, d, *J* 9.4, 3-H), 6.37 (1H, dd, *J* 3.2, 1.9, ArH), 6.32 (1H, dd, *J* 3.2, 0.7, ArH), 5.76 (1H, bs, NH), 4.66 (2H, d, *J* 5.4, CH_2); δ_C (75 MHz; $CDCl_3$) 160.7 (C), 150.6 (C), 146.7 (CH), 142.6 (CH), 136.3 (C), 133.0 (CH), 110.6 (CH), 107.9 (CH), 39.1 (CH_2).

5-Nitro-2-(pyrrolidin-1-yl)pyridine 46

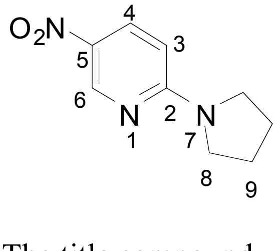

The title compound was synthesised following general procedure method A from 2-chloro-5-nitropyridine (277 mg, 1.75 mmol), pyrrolidine (144 μL, 1.75 mmol) and potassium fluoride

(203 mg, 3.50 mmol) in water (1 mL) at 100 °C for 17 h to give the *title compound* as a yellow crystalline solid (250 mg, 74%); mp 121-123 °C; (Found; MH^+, 194.0932. $C_9H_{12}N_3O^+$ requires 194.0924); ν_{max} ($CDCl_3$)/cm^{-1} 2980, 2874, 1599, 1571, 1522, 1485; δ_H (400 MHz; $CDCl_3$) 8.99 (1 H, d, *J* 2.5, 6-H), 8.11 (1 H, dd, *J* 9.4, 2.5, 4-H), 6.27 (1 H, d, *J* 9.4, 3-H), 3.67 (2 H, bs, 8-H), 3.41 (2 H, bs, 8-H), 2.05 (4 H, bs, 9-H); δ_C (75 MHz; $CDCl_3$) 158.7 (C), 147.0 (CH), 134.4 (C), 132.3 (CH), 105.2 (CH), 47.4 (CH_2), 25.3 (CH_2).

4-(5-Nitropyridin-2-yl)morpholine 47

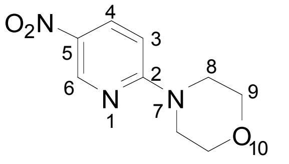

The title compound was synthesised following general procedure method A from 2-chloro-5-nitropyridine (277 mg, 1.75 mmol), morpholine (153 µL, 1.75 mmol) and potassium fluoride (203 mg, 3.50 mmol) in water (1 mL) at 100 °C for 17 h to give the *title compound* as a yellow solid (319 mg, 87%); mp 136-138 °C; (Found; MH^+, 210.0880. $C_9H_{12}N_3O_3^+$ requires 210.0873); ν_{max}/cm^{-1} ($CHCl_3$) 3011, 2862, 1597, 1578, 1510, 1449; δ_H (400 MHz; $CDCl_3$) 9.07 (1H, d, *J* 2.7, 6-H), 8.26 (1H, dd, *J* 9.5, 2.7, 4-H), 6.59 (1H, d, *J* 9.5, 3-H), 3.85-3.83 (4H, m, CH_2), 3.79-3.76 (4H, m, CH_2); δ_C (75 MHz; $CDCl_3$) 160.5 (C), 146.3 (CH), 135.4 (C), 133.1 (CH), 104.5 (CH), 66.5 (CH_2), 45.1 (CH_2).

1-(5-Nitropyridin-2-yl)-4-phenylpiperazine 48

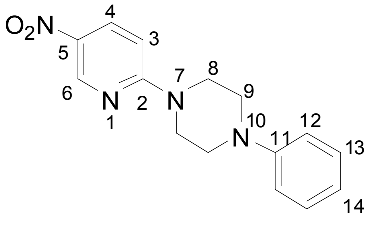

The title compound was synthesised following general procedure method A from 2-chloro-5-nitropyridine (277 mg, 1.75 mmol), 1-phenylpiperazine (276 μL, 1.75 mmol) and potassium fluoride (203 mg, 3.50 mmol) in water (1 mL) at 100 °C for 17 h to give the *title compound* as a brown solid (380 mg, 76%); mp 154-156 °C; (Found; MH^+, 285.1337. $C_{15}H_{17}N_4O_2^+$ requires 285.1346); ν_{max}/cm^{-1} ($CHCl_3$) 3008, 1595, 1577, 1494, 1452, 1429; δ_H (400 MHz; $CDCl_3$) 9.09 (1H, d, *J* 2.7, 6-H), 8.26 (1H, dd, *J* 9.5, 2.7, 4-H), 7.35-7.30 (2H, m, Ar-H), 7.00-6.92 (3H, m, Ar-H), 6.64 (1H, d, *J* 9.5, 3-H), 3.97 (4H, t, *J* 5.1, CH_2), 3.35 (4H, t, *J* 5.1, CH_2); δ_C (75 MHz; $CDCl_3$) 160.3 (C), 150.7 (C), 146.5 (CH), 135.2 (C), 133.1 (CH), 129.3 (CH), 120.5 (CH), 116.4 (CH), 104.6 (CH), 49.2 (CH_2), 44.8 (CH_2).

***N*-(4-Methoxyphenyl)-5-nitropyridin-2-amine 49**

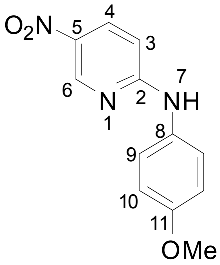

The title compound was synthesised following general procedure method A from 2-chloro,5-nitropyridine (277 mg, 1.75 mmol), *p*-anisidine (218 mg, 1.75 mmol) and potassium fluoride (203 mg, 3.50 mmol) in water (1 mL) at 100 °C for 17 h to give the *title compound* as a orange solid (315 mg, 73%); mp 158-160 °C; (Found; MH^+, 246.0873. $C_{12}H_{12}N_3O_3^+$ requires 246.0873); ν_{max}/cm^{-1} ($CHCl_3$) 3402, 3009, 2839, 1713, 1599, 1509; δ_H (400 MHz; $CDCl_3$) 9.07 (1H, d, *J* 2.6, 6-H), 8.21 (1H, dd, *J* 9.4, 2.6, 4-H), 7.45 (1H, bs, NH), 7.29 (2H, d, *J* 8.9, ArH), 6.98 (2H, d, *J* 8.9, ArH), 6.62 (1H, d, *J* 9.4, 3-H), 3.86 (3H, s, CH_3); δ_C (75 MHz; $CDCl_3$) 160.6 (C), 158.0 (C), 146.8 (CH), 136.7 (C), 133.4 (CH), 130.4 (C), 125.7 (CH), 115.0 (CH), 105.6 (CH), 55.6 (CH_3).

References

[1] M. G. Organ, M. Abdel-Hadi, S. Avola, I. Dubovyk, N. Hadei, E. A. B. Kantchev, C. J. O'Brien, M. Sayah, C. Valente, *Chem. Eur. J.* **2008**, *14*, 2443-2452.

[2] T. H. Graham, W. Liu, D.-M. Shen, *Org. Lett.* **2011**, *13*, 6232-6235.

[3] D. S. Ermolat'ev, E. V. Van der Eycken, *J. Org. Chem.* **2008**, *73*, 6691-6697.

[4] C. V. Reddy, J. V. Kingston, J. G. Verkade, *J. Org. Chem.* **2008**, *73*, 3047-3062.

[5] B. M. Choudary, C. Sridhar, M. L. Kantam, G. T. Venkanna, B. Sreedhar, *J. Am. Chem. Soc.* **2005**, *127*, 9948-9949.

[6] J. S. Siddle, A. S. Batsanov, M. R. Bryce, *Eur. J. Org. Chem.* **2008**, 2746-2750.

[7] Z. Shen, X. He, J. Dai, W. Mo, B. Hu, N. Sun, X. Hu, *Tetrahedron* **2011**, *67*, 1665-1672.

[8] N. K. Lee, J. W. Lee, S. Lee, G.-J. Im, H. Y. Han, T. K. Kim, Y. H. Kim, W.-J. Kwak, S. W. Kim, J. Ha, E. K. Kim, J. K. Lee, C. Y. Yoo, D. Y. Lee, **2006**, WO 2006/071095 A1.

[9] B. E. Christensen, B. Graham, A. J. Tomisek, *J. Am. Chem. Soc.* **1946**, *68*, 1306-1308.

[10] G. E. Hardtmann, F. Park, H. Ott, **1969**, US 3,470,182.

[11] D. Maiti, B. P. Fors, J. L. Henderson, Y. Nakamura, S. L. Buchwald, *Chem. Sci.* **2011**, *2*, 57-68.

[12] B. J. Tardiff, M. Stradiotto, *Eur. J. Org. Chem.* **2012**, 3972-3977.
